# Impact of maternal posture on fetal physiology in human pregnancy: a narrative review

**DOI:** 10.3389/fphys.2024.1394707

**Published:** 2024-05-17

**Authors:** Allan J. Kember, Jennifer L. Anderson, Sarah C. House, David G. Reuter, Craig J. Goergen, Sebastian R. Hobson

**Affiliations:** ^1^ Temerty Faculty of Medicine, Department of Obstetrics and Gynaecology, University of Toronto, Toronto, ON, Canada; ^2^ Shiphrah Biomedical Inc., Toronto, ON, Canada; ^3^ Larner College of Medicine, University of Vermont, Burlington, VT, United States; ^4^ Temerty Faculty of Medicine, Medical Education, University of Toronto, Toronto, ON, Canada; ^5^ Cardiac Innovations, Seattle Children’s Hospital, Seattle, WA, United States; ^6^ Weldon School of Biomedical Engineering, Purdue University, West Lafayette, IN, United States; ^7^ Temerty Faculty of Medicine, Institute of Medical Science, University of Toronto, Toronto, ON, Canada; ^8^ Maternal-Fetal Medicine Division, Mount Sinai Hospital, Toronto, ON, Canada

**Keywords:** gravity, posture, maternal, pregnancy, obstetrics, physiology, pathophysiology

## Abstract

In numerous medical conditions, including pregnancy, gravity and posture interact to impact physiology and pathophysiology. Recent investigations, for example, pertaining to maternal sleeping posture during the third trimester and possible impact on fetal growth and stillbirth risk highlight the importance and potential clinical implications of the subject. In this review, we provide an extensive discussion of the impact of maternal posture on fetal physiology from conception to the postpartum period in human pregnancy. We conducted a systematic literature search of the MEDLINE database and identified 242 studies from 1991 through 2021, inclusive, that met our inclusion criteria. Herein, we provide a synthesis of the resulting literature. In the first section of the review, we group the results by the impact of maternal posture at rest on the cervix, uterus, placenta, umbilical cord, amniotic fluid, and fetus. In the second section of the review, we address the impact on fetal-related outcomes of maternal posture during various maternal activities (e.g., sleep, work, exercise), medical procedures (e.g., fertility, imaging, surgery), and labor and birth. We present the published literature, highlight gaps and discrepancies, and suggest future research opportunities and clinical practice changes. In sum, we anticipate that this review will shed light on the impact of maternal posture on fetal physiology in a manner that lends utility to researchers and clinicians who are working to improve maternal, fetal, and child health.

## Introduction

Maternal posture and pregnancy are intimately related and their interactions have important impacts on pregnancy health outcomes. As such, our group has a comprehensive review on the interactions between maternal posture and maternal physiology and pathophysiology at rest during pregnancy currently under review^1^, which is the complement of the current review. In the current review, we extend this work to synthesize the international literature describing the impact of maternal posture on fetal physiology and pathophysiology. Building on the premises that posture and gravity interact to alter the physiology of organ systems across medical specialties, and that these interactions exist in almost every maternal organ system during pregnancy in obstetrics, we embark on this review with the hypothesis that the utero-feto-placental unit must also be impacted by maternal posture. A landmark publication by Stacey et al. in 2011 demonstrated an association between maternal supine sleep and late stillbirth that initially prompted wide international interest in this subject (Stacey et al., 2011). Indeed, the aim of this review is to further ignite the research and clinical communities to continue exploring this important topic. The purpose of this review is to both present the international body of literature to date and highlight knowledge gaps and opportunities for future work.

The current review spans conception, pregnancy, and postpartum, and we deliberately employ the term “posture” because its meaning has a closer association with the human body than “position.” We also consider healthy pregnancies as well as those with comorbidities, with a particular focus on impact of maternal posture on fetal physiology at rest and during various maternal activities. We aim for this review to be relevant to the everyday lives of researchers and practicing clinicians and their patients; however, we hope it is of interest to an even larger audience. As space travel and habitation become a tangible aspiration for humans, the question of reproduction and pregnancy in sub-gravitational environments necessarily ensues, to which our review also has relevance.

## Methods

**FIGURE 1:**
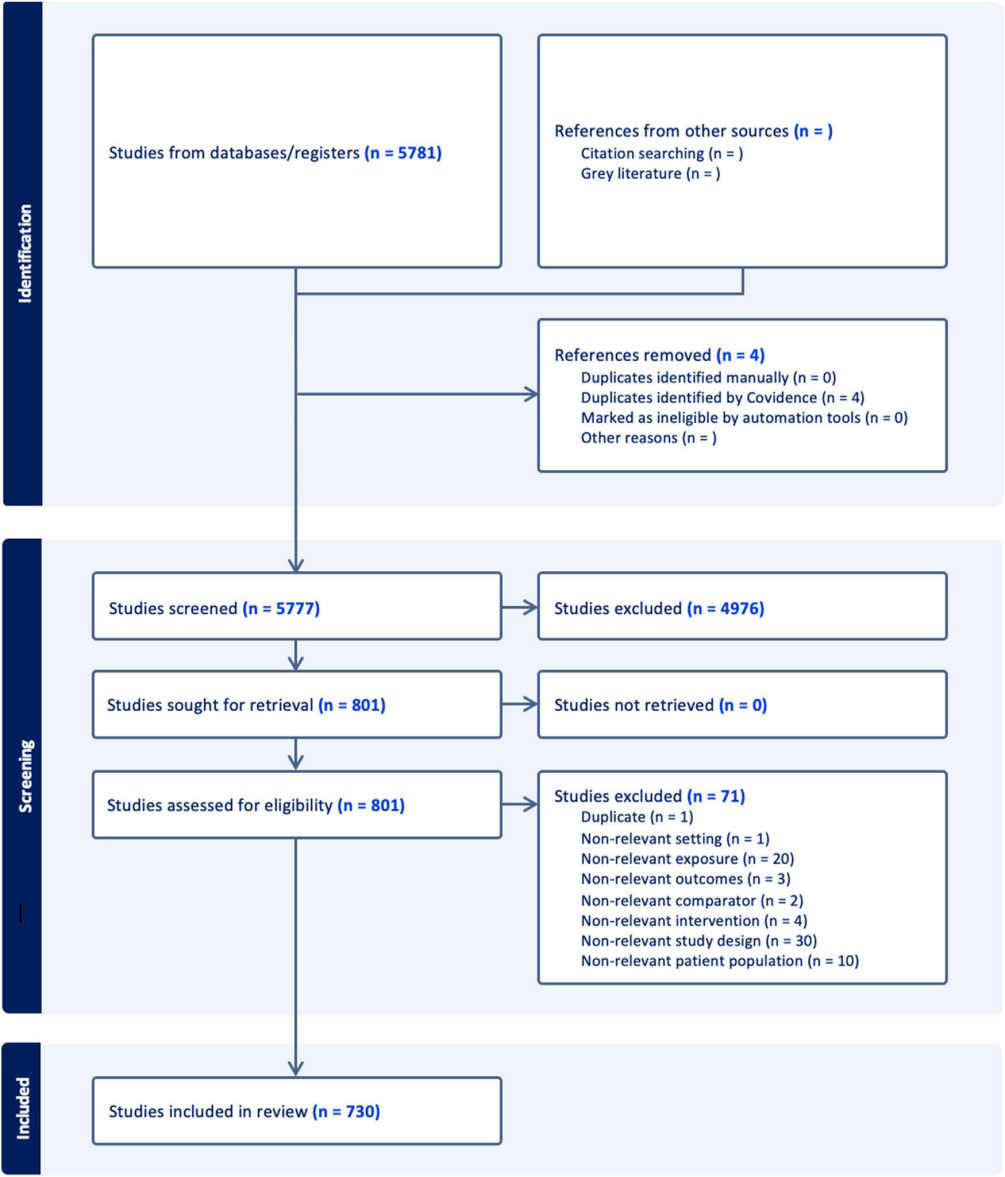
PRISMA flow diagram outlining the study selection process.

A comprehensive literature search was performed in August 2021 using MEDLINE. The keywords either described pregnancy or body posture (see Supplementary material Data Sheet 1—Search Strategy Keywords). The search was narrowed to results published between 1 January 1991 through 8 August 2021. Results were filtered to English language and human participants using MEDLINE’s built-in filters. The search yielded 5,781 publications. Four duplicates were removed. Search results were then independently screened by four reviewers for adherence to inclusion and exclusion criteria. Included papers involved participants in the conception, antepartum, intrapartum, and/or postpartum periods and investigated the effect of maternal body posture on a maternal or fetal organ system or physiological/pathophysiological process. We excluded book chapters, editorials, commentaries, paper replies or responses, reviews (with the exception of systematic, meta-analysis, and Cochrane reviews), or if the publications were related to male fertility or infertility. This further refined the selection to a final number of 730 studies (Figure 1). Of the 730 studies, 242 included the cervix, uterus, placenta, umbilical cord, amniotic fluid, and fetus and are the subject of this review.

## Results

Research articles pertaining to the impact of maternal posture on the cervix, uterus, placenta, umbilical cord, amniotic fluid, and fetus can be generally divided into 1) those that consider the impact of maternal posture at rest and 2) those that consider the impact of maternal posture during various activities. As such, we present these aspects separately. Furthermore, we organize the former category of results based on general obstetric entities—beginning with the cervix, followed by the uterus, placenta, umbilical cord, amniotic fluid, and finally, the fetus.

## Cervix

Maternal posture can influence cervical shape and mechanics during pregnancy; however, since the majority of studies relating to this were primarily focused on the impact of posture during maternal activities and medical procedures, we have reserved their discussion to the Maternal Activities and the Medical Procedures sections.

## Uterus

The effect of posture and gravity on the gravid uterus, particularly vascular hemodynamics, has been studied for several decades. We commence our discussion with the literature relating to the effects of maternal posture on uterine hemodynamics and follow this with examination of uterine contractions.

### Hemodynamics

Uterine vascular hemodynamics are key to a healthy pregnancy. In a study of 30 participants, Jaffa *et al.* did not find a difference in the systolic/diastolic ratio (S/D) of the uterine arteries (UtA) in the supine position (assessed 5 minutes following adoption of the position) compared with the lateral position between 35–40 weeks gestation (Jaffa et al., 2001). Note that an unchanged S/D ratio does not mean that resistance is unchanged; rather, it means that the resistance in systole and diastole have both not changed or have both changed proportionally such that the relative amplitude of the systolic velocity compared to the diastolic velocity is unchanged. An earlier and much larger (*n* = 90) study, Ryo et al. found an elevated UtA resistance index (RI) in association with the degree of inferior vena cava (IVC) compression in the supine position (assessed 5 minutes following adoption of the position) (Ryo et al., 1996). The RI is a more direct reflection of the actual resistance of the distal placental capillary bed to forward blood flow. Park and Hikada’s results are concordant, demonstrating increased UtA resistance when supine compared to when in the left lateral posture (Park and Hidaka, 1991). Of note, the findings of Ryo et al. were observed at 24–27 weeks (Ryo et al., 1996), which indicate that the effects of supine posture on uterine hemodynamics occur at least by the late second trimester. In the third trimester, Jeffreys et al. observed that blood flow in the right UtA is reduced by 35% and 13% during supine rest and supine exercise, respectively, compared to its value in the left lateral posture, and this correlated directly with birthweight and placenta weight at birth (Jeffreys et al., 2006). Their results also corroborate both Jaffa et al. and Ryo et al.’s—they observed an unchanged S/D ratio and increased resistance when supine compared to left lateral posture (Ryo et al., 1996; Jaffa et al., 2001). Most recently, in the supine posture at 34–38 weeks, Couper et al. utilized functional MRI and observed 27.0% and 19.0% reductions in left and right internal iliac artery blood flow, respectively, in comparison to the lateral position (Couper et al., 2021). Note that each internal iliac artery gives rise to the ipsilateral UtA and are the main blood supply of the uterus and placenta.

### Contractions

“Orthostatic uterovascular syndrome” (OUS), first described by Schneider et al, (1984), describes a condition where, when standing motionless and unsupported in the third trimester, the maternal heart rate (HR) is observed to undergo cyclic accelerations. The physiology behind OUS is thought to be that the gravid uterus compresses the pelvic vessels when standing, reducing blood flow velocity in the femoral vein, which is followed by maternal HR acceleration (compensating for reduced cardiac return and preload) and a spontaneous uterine contraction, which is followed by increased blood flow velocity in the femoral vein and normalization of the maternal HR (Schneider and Deckardt, 1991; Schneider et al., 1993). The maternal HR normalizes within 14 s following the beginning of a uterine contraction, and this in-phase occurrence strengthens our current understanding of OUS physiology (Schneider et al., 1993). Furthermore, serum catecholamines are known to increase in response to physical stress, such as the upright posture, and are also known to decrease uterine blood flow (Katz et al., 1991), which may be an additional contributing mechanism to OUS. In order to be classified as OUS, the maternal HR must have a regular occurrence of accelerations (3 accelerations in 10 min) with an amplitude of at least 15 beats/min above the baseline. In one study of uncomplicated pregnancies between 36–41 weeks, OUS was observed to occur in 33 of 55 (60%) participants (Schneider et al., 1984), and this agrees with another study by the same authors a decade later in which prevalence of OUS was observed to be 5% at 24 weeks’ gestation and reached a peak of 71% at 38 weeks’ gestation (Schneider et al., 1993). Furthermore, as gestation advances, there is a three-fold increase in the frequency of uterine contractions when upright in OUS compared to those occurring in the left lateral posture. The frequency of upright uterine contractions in OUS may be reduced by 20% by leaning forward slightly, bending at the waist. Eighty-percent of these contractions are not felt by the patient, yet hemodynamic effects, primarily in the maternal HR, can be observed. Despite these cyclic fluctuations in maternal HR, maternal blood pressure (BP) remains relatively stable with no change in the diastolic BP and a 1.4% reduction in the systolic BP at the peak of the maternal HR acceleration (Schneider et al., 1993). However, roughly 25% of fetuses experience a reduction in fetal HR variability during OUS, suggesting a fetal effect due to maternal posture-related hemodynamic changes (Schneider et al., 1984).

Additionally, Park and Hidaka demonstrated a significant reduction in the frequency and intensity of uterine contractions in the left lateral posture compared to supine, and this correlated directly with maternal cardiac output (CO) (Park and Hidaka, 1991). They attribute this physiology to compression of the IVC by the gravid uterus when supine and resulting hemodynamic effects similar to that seen in OUS, namely, lower extremity venous congestion, resultant maternal HR acceleration, a spontaneous uterine contraction, and restoration of maternal cardiac preload and output, followed by normalization of maternal HR. This cyclic pattern of maternal HR reductions following uterine contractions was also observed in 11% of participants in a study of 119 low-risk, non-labouring, pregnancies at term (Ibrahim et al., 2015); however, the cyclic changes in maternal HR occurred in both supine and non-supine horizontally-oriented postures, being mainly associated with uterine activity, meant that no impact on the fetal HR pattern was observed. In term, non-laboring pregnancies, Ibrahim *et al.* found that the periodic maternal HR changes due to uterine activity are significantly more common (85% vs. 11%) when in the right lateral posture compared to the left (Ibrahim et al., 2015). It is important to note that this physiology is similar to that observed in supine hypotensive syndrome (SHS), which we discuss fully in our review of maternal posture-physiology interactions. As for clinical implications of posture-related uterine contractions, beyond left-uterine displacement or shifting to a non-supine posture being a universally recommended component of intrauterine resuscitation, we found one case report suggesting that in pregnancies that are sensitive to changes in intravascular volume (e.g., aortic stenosis), avoidance of the supine posture may attenuate the impact of uterine contractions (and hence autotransfusion of approximately 400 mL into maternal central circulation with each contraction) on maternal hemodynamics during labor (Baird and Arkoosh, 2012).

Overall, these reports suggest that compression of venous circulation by the gravid uterus, whether standing upright or in the supine posture, results in reduced maternal cardiac preload, which may precipitate uterine contractions in an effort to restore preload. The mechanism behind this is unelucidated and a potential area of future research.

## Placenta and umbilical cord

We combine our discussion of the umbilical cord with the placenta because many physiologic parameters measured in the umbilical cord clinically (e.g., umbilical artery pulsatility index) are a reflection of the health and function of the placenta. That said, we acknowledge that some parameters of the umbilical cord (e.g., umbilical vein pulsations) are a reflection of fetal health. We observed that the literature vis-à-vis effects of maternal posture on the placenta and cord could be categorized into two major areas of study—hemodynamics and oxygenation. As such, we divide our approach to this section into these two categories.

### Hemodynamics

Jaffa *et al.* (*N* = 30) did not find a difference in the S/D ratio of the umbilical arteries (UA) in the supine posture (assessed 5 minutes following adoption of the position) compared with the lateral posture between 35–40 weeks gestation (Jaffa et al., 2001). These findings are corroborated by Silva *et al.* (*N* = 20) who were unable to demonstrate differences in the UA pulsatility index (PI) and UA S/D between the left lateral and supine postures (assessed 5 minutes after assuming the lateral posture and at five and 10 minutes after assuming the supine posture) between 36–40 weeks (Silva et al., 2017). These studies were small but are in harmony with the findings of Ryo *et al.* (*N* = 90), who were unable to demonstrate an effect of maternal supine posture on UA flow parameters (assessed 5 minutes following adoption of the posture) at 24–27 weeks (Ryo et al., 1996).

Khatib *et al.*, however, investigated pregnancies between 36–40 weeks (*N* = 23) and found that UA PI and UA S/D both increased (*p* < 0.001) after changing to the supine posture from the left lateral posture (assessed 15 minutes following adoption of each posture), reflecting increased umbilical-placental vascular resistance to forward flow when supine (Khatib et al., 2014). Nakai et al*.* (*N* = 23) had similar results in pregnancies after 34 weeks where they demonstrated that the UA S/D’s were significantly lower in the prone posture (*p* < 0.01) and left lateral posture (*p* = 0.055) compared to the supine posture (Nakai et al., 1998). Similarly, in a study in pregnancies between 23–36 weeks gestation, van Katwijk and Wladimiroff (*N* = 27) found that the UA PI increased significantly upon maternal change from standing to the supine posture and decreased significantly upon maternal change from the supine posture to standing (assessed 2–4 min following adoption of the posture), a phenomenon that the authors attributed to the “sluice flow” effect (van Katwijk and Wladimiroff, 1991). Sorensen *et al.* demonstrated a significant increase in the UA S/D ratio in the upright posture compared to the left lateral posture in pregnancies with hypertension but not in pregnancies with normotension; however, they did not include the supine posture in their protocol, which makes it challenging to contextualize these results (Sorensen et al., 1992).

In another study by Ryo et al*.* (*N* = 202), an abnormal UA RI, defined as being greater than the 90th percentile, in the supine posture at 35–37 weeks was found to have a sensitivity and positive predictive value (PPV) of 53% and 43%, respectively, for subsequent abnormal outcomes including small-for-gestational-age (SGA), fetal distress, and pregnancy-induced hypertension. Sensitivity and PPV declined precipitously for an abnormal UA RI in the left lateral posture at the same gestation and for both postures at an earlier gestation (27–29 weeks). These findings may lend support to clinical use of the supine posture in late pregnancy combined with UA Doppler velocimetry as a “stress test” for identifying at-risk pregnancies (Ryo et al., 1998).

Discrepancy in the aforementioned studies may be due to methodological differences. That said, while all the studies generally described their methods for obtaining Doppler measurements, the older studies provided less information than the newer studies. Most reported the ultrasound manufacturer, probe type and frequency, number of sonographers, timing and averaging of multiple measurements, and performing measurements in the absence of fetal body and breathing movements, with slight differences in these parameters, but only one study reported specifics of the criteria used in obtaining the measurements (e.g., keeping the insonation angle as close to zero as possible). Furthermore, most studies attempted to minimize variability by using one sonographer to make all the measurements. We submit, however, that differences and variability in anatomy and physiology between individuals may also contribute to the discrepancy seen in the literature. Similar to how some pregnancies are affected by SHS and some are not, it is possible that a supine-related increase in placental resistance follows the same individual variation. Such duality might be a result of various other contributing and confounding factors that impact the immediate uteroplacental environment and should be accounted for in future research. For example, polyhydramnios, supine posture, obesity, and sleep apnea all increase intra-abdominal pressure. Insightful *in vitro* models have been developed for states of increased external pressure on the placenta (Karimu and Burton, 1994; Kranenburg-Lakeman et al., 2001). Polyhydramnios is a state of increased pressure on the placenta from the fetal side, and SHS is a state of increased pressure on the placenta from the maternal side via increased venous pressures in the intervillous space from downstream occlusion of the IVC by the gravid uterus in the maternal supine posture. These models have demonstrated that fetal-placental capillaries are elastic, deformable, and compressible (Karimu and Burton, 1994), and these capillaries have nonlinear perfusion pressure-dependent resistance (Kranenburg-Lakeman et al., 2001). Notably, increased external pressures on the placenta increases placental bed resistance as seen by the fetus but only at the lower side of the pressure-flow curve (Kranenburg-Lakeman et al., 2001). That is, at lower perfusion pressures (such as would occur during fetal hypotension), external pressure on the fetal vessels in the placenta may overcome the intraluminal pressure of some of the fetal vessels, collapsing them, and causing the placental bed resistance to forward flow to increase. This may indicate that fetuses prone to hypotension are more vulnerable to the mechanical effects of external pressure on the placenta such as that encountered in polyhydramnios or SHS, which may occur, for example, in a fetus with a cardiac arrhythmia and duodenal atresia.

Indeed, Zun et al. recently employed advanced MRI techniques showing that fetuses with congenital heart disease are particularly affected by maternal posture as demonstrated by significantly lower global placental perfusion in the supine posture compared to the lateral side-lying posture (Zun et al., 2017). It is likely that compression of the placental capillaries occurs when the pressure in the intervillous space (maternal side) exceeds the pressure in the capillary lumen (fetal side), which results in an increase in the capillary resistance—recall that for pipe flow, resistance to flow is inversely proportional to the fourth power of the vessel’s radius by Poiseuille’s equation (Karimu and Burton, 1994). Overall, this effect may also contribute to the variable changes seen in placental resistance and UA flow parameters in the maternal supine posture.

### Oxygenation

In a recent study employing functional MRI at 34–38 weeks, Couper *et al.* found that maternal supine posture results in a 6.2% reduction in oxygen transfer across the placenta (*p* = 0.038), an 11.2% reduction trend in oxygen delivery to the fetus (*p* = 0.060), and a 4.4% reduction trend in fetal oxygen saturation (*p* = 0.079) in comparison to the lateral posture (Couper et al., 2021). Corroborating these findings, Abaci Turk *et al.* found significantly larger increases in global placental R2* (a reflection of blood oxygen saturation levels) in the maternal supine posture compared to the left lateral posture with maternal hyperoxygenation via 100% fraction of inspired oxygen (FiO_2_) non-rebreather face mask for 5 minutes using a technique called BOLD (blood-oxygen-level-dependent) MRI in uncomplicated pregnancies between 27–36 weeks (Abaci et al., 2020). The authors suggested these results were most consistent with the sigmoidal nature of the oxygen-hemoglobin dissociation curve: for a given change in the partial pressure of oxygen (pO_2_), there is a greater absolute change in oxy-hemoglobin percent saturation when starting at a lower pO_2_ (when supine) than when starting at a higher pO_2_ (when non-supine). Furthermore, it is well known that maternal arterial pO_2_ is consistently lower in the supine posture than in the left lateral posture (Dunietz et al., 2020). Taken together, this indicates a significantly lower blood oxygen saturation in the placenta and umbilical cord in the maternal supine posture compared to the left lateral posture.

## Amniotic fluid

In the third trimester, 1 hour of maternal rest in the left lateral posture has been found to significantly increase the fetal urine production rate, approximately doubling it, as compared to the semi-supine posture (Ülker et al., 2011). Given that the main source of amniotic fluid in the third trimester is fetal urine, this finding is corroborated by multiple studies demonstrating that maternal rest in the left lateral posture in the third trimester increases the amniotic fluid index (AFI) by about 2 cm (from 15.1 cm to 17.4 cm) (Ülker et al., 2011; Ulker et al., 2012a; Ulker et al., 2012b; Ülker and Çiçek, 2013). Ülker and others found that the greatest increase in amniotic fluid volume (AFV) occurs in the first 15-min interval, followed by the second greatest increase in the second 15-min interval, and the AFV continues to trend upwards, albeit at slower rates, across subsequent 15-min intervals for at least 90 min, after which it likely plateaus. Corroborating this finding, Ülker and Çiçek demonstrated, interestingly, that 15 minutes of rest in the right lateral posture decreases the AFV in comparison to the left lateral position for the same amount of time, but this does not change the AFV in comparison to 15 minutes of rest in the semi-recumbent supine posture (Ülker and Çiçek, 2016). The clinical implications of these findings are yet to be elucidated.

In pregnancies with normal AFI in the third trimester, compared to the supine recumbent posture, the AFI in the supine position with 45 of upper body elevation is not changed when immediately measured (the only time elapsed between measurements was time taken to adjust maternal posture) (Tressler et al., 2006). Therefore, supine recumbency is not required for AFI measurements, which has obvious benefits for patients with SHS or similar conditions who experience unpleasant symptoms in as little as 3 minutes after assuming the supine posture.

## Fetal

Gravitational force in the intrauterine environment is essential for fetal muscle, bone, and cardiovascular development in the second half of pregnancy. At 15 weeks’ gestation, the AFV-to-fetal-volume ratio is 3:1, and this decreases to 0.17:1 at term (Sekulić et al., 2005). The buoyant forces of the amniotic fluid being displaced by the floating fetal volume interact with gravitational forces acting on the fetal mass according to Archimedes’ principle and induce a near neutral condition similar to weightlessness up to about 21–22 weeks’ gestation. As fetal development progresses beyond 26 weeks’ gestation, the increasing specific gravity of the fetal volume, increasing fetal mass, and decreasing AFV-to-fetal-volume ratio results in a relative decrease in buoyant force and relative increase in gravitational force. The fetal apparent weight becomes approximately 60%–80% of its actual weight. If it were not for these changes, the fetus would suffer a range of deconditioning effects including skeletal and myocardial muscle hypotrophy, reduction in myosin heavy chain type I fibers in the extensors, and osteopenia and hypoplasia of the vertebrae and long bones (Sekulić et al., 2005).

In this section, we focus our discussion on the impact of maternal posture on fetal heart rate, fetal cerebral circulation, fetal behavioral state, and fetal growth.

### Fetal heart rate

The effect of maternal posture on fetal heart rate (FHR) is debatable and the literature has not reported consistent results, largely related to differing study methodology. Indeed, a recent Cochrane review on cardiotocography (CTG) (assessment of FHR pattern and its temporal relationship to uterine contractions) by Alfirevic et al*.* urged future research to address the potential contribution of maternal supine posture to adverse outcomes and whether maternal posturing could be used to reduce adverse neonatal outcomes (Alfirevic et al., 2017). Regarding studies investigating maternal posture and the FHR in the third trimester in singleton pregnancies with intact membranes, we found that six of these studies were large, with over 100 participants in each study. Of these, one reported that posture makes no difference, one reported that supine posture was favorable, and four reported that supine posture was unfavorable. We discuss these six reports first followed by several smaller studies.

In a randomized controlled trial (RCT), Moffat and van den Hoff (*N* = 573) reported no significant difference in the incidence of non-reactive CTG and time to CTG reactivity (using the Dawes-Redman criteria) between the three postures they studied: semi-recumbent posture (semi-sitting with the upper body elevated at 30) with 45 left lateral tilt (wedge placed under the right hip and pelvis), with a 45 right lateral tilt, or with no tilt (Moffatt and van den Hof, 1997).

Nathan et al*.* (*N* = 108) completed a study in which they performed two 10-min CTG’s in two different postures: 1) semirecumbent (elevation of upper body not specified) and 2) left lateral recumbent (Nathan et al., 2000). Independent of order, there was more 10-min CTG non-reactivity in the left lateral recumbent posture than in the semirecumbent position. On a logistic regression, the first 10-min CTG and the left lateral recumbent starting posture independently predicted non-reactive CTG (Nathan et al., 2000). We find these results, namely, that the supine posture was favorable and the left lateral posture was unfavorable, to be paradoxical and less helpful since the CTG duration used in this study is not typically used in clinical practice.

Tamás et al*.* (*N* = 106) provided unique insight into FHR tracing morphology. The authors demonstrated a statistically significant increase in the number of FHR accelerations (25%) and overall and short-term FHR variation (20% each) and a decrease in basal FHR (3.5%) with the left lateral posture in comparison to supine (Tamás et al., 2007). There was no difference in FHR decelerations between the postures. Significant correlations were found between FHR short term variation and maternal CO in the supine posture and between the number of FHR accelerations and maternal stroke volume and CO in the left lateral posture. When maternal posture changed (from supine to left lateral), the resulting change in stroke volume (12% increase) correlated significantly with FHR overall variation and short term variation. Aluş et al*.* (*N* = 408) added to this work by considering more maternal postures (Aluş et al., 2007). They randomly assigned participants to complete a 20-min CTG in one of four postures: 1) seated upright, 2) semi-fowler, 3) supine, and 4) supine-left. The CTG reactivity (≥2 fetal movements associated with a ≥15 bpm acceleration lasting ≥15 s) was significantly lower when supine (69%) compared to any of the three other postures. The semi-fowler posture yielded the highest CTG reactivity (85.3%). Similar to Moffat and van den Hoff (Moffatt and van den Hof, 1997), Aluş *et al.* did not find a significant difference in the time taken to reach CTG reactivity between the four maternal postures. Cito et al. (*N* = 368) highlighted the importance of factoring in the effect of gestational age when investigating the impact of maternal posture on FHR (Cito et al., 2005). In their study, the time taken to achieve a reactive CTG (defined as three large FHR accelerations) was significantly decreased as pregnancy advanced (from <37 weeks, 37–39 weeks, and >39 weeks) when the CTG was completed during sitting or walking (Cito et al., 2005). This time, however, was unchanged with advancing pregnancy when the CTG was completed in the reclining posture. After 39 weeks, there were significantly more FHR variable decelerations in the reclining posture when compared to sitting and walking. Finally, and most recently, Ibrahim et al*.* (*N* = 118) reported increased FHR, accelerations, and fetal movements in the left lateral posture followed by semi-fowler, compared to supine (Ibrahim et al., 2021). No differences, however, were found between postures for FHR variability and CTG reactivity.

A small but unique study by Ellington et al. (*N* = 25) reported no significant differences in FHR (nor UA flow morphology) between the various tilted postures they tested including 1) supine, 2) supine with 5 (operating table angle) right lateral tilt, 3) supine with 10° right lateral tilt, 4) supine with 5° left lateral tilt, 5) supine with 10° left lateral tilt, and 6) supine with 10° left lateral tilt with a 10 cm wide wedge (rolled blanket) placed under the right hip (Ellington et al., 1991). Their study was completed in pregnancies between 25–40 weeks, however, which may have diluted the impact of compressional effects seen at more advanced gestations. Furthermore, others have shown that 15° of left lateral tilt is required to relieve physiologic effects of aortocaval compression by the gravid uterus and restore maternal CO (Lee et al., 2012). Armstrong et al. (*N* = 25) also reported null findings regarding the effect of maternal posture on FHR (and UA PI) in non-laboring, uncomplicated, term pregnancies (Armstrong et al., 2011). They considered four postures: supine with 15° left lateral tilt, sitting with neck and hips flexed, left lateral, and right lateral. Dennis et al. (*N* = 30) also found that maternal posture did not affect FHR in their sample of non-laboring, uncomplicated, term pregnancies (Dennis et al., 2018a). They considered three postures: 1) left lateral decubitus, 2) ramped upper-body with left lateral pelvic tilt (wedge), and 3) ramped upper body alone. Of note, they also found no difference in maternal CO in each posture, so a null effect of these maternal postures on FHR is not unexpected.

Swansburg *et al.* investigated pregnancies with preeclampsia (*N* = 9) at 32–40 weeks and used normotensive pregnancies (*N* = 18) as comparators (Swansburg et al., 2005). When presented with orthostatic stress (i.e., from lying to standing), parasympathetic tone decreased in both groups, but the normotensive group had more spontaneous FHR accelerations. These results indicate that fetuses in pregnancies with preeclampsia may be affected during orthostatic challenges, and this effect could be a result of autonomic dysfunction coupled with intravascular volume contraction seen in preeclampsia.

Finally, we found two studies reporting on FHR with regard to prone posture. Dennis et al. studied pregnancies with preeclampsia (*N* = 15) and healthy normotensive comparators (*N* = 50) at term (Dennis et al., 2018b). They found no difference in FHR in the prone posture (resting on a specially designed pillow with a center hole that supports the gravid uterus) compared with the left lateral posture. Note, however, that they did not actually measure the FHR when the participant was prone and only measured it immediately after the participant returned to the left lateral posture after resting in the prone posture for 5 minutes. Oliveira et al. (*N* = 33) also reported no differences in FHR between prone (resting for 6 minutes on a specially designed stretcher with a center hole allowing the gravid uterus to pass through it) and non-prone postures (included fowlers, supine, and left lateral) (Oliveira et al., 2017).

Overall, the literature on FHR suggests that it is impacted by maternal posture, specifically the supine posture seems to be unfavorable. More work, however, remains to be done as complete consensus has not yet been reached.

### Fetal cerebral circulation

Fewer studies reported on fetal cerebral circulation, but blood flow in the brain is clearly an integral part of fetal neurological development. Khatib et al*.* (*N* = 23) studied low-risk pregnancies at 36–40 weeks and found that the middle cerebral artery (MCA) PI and the MCA peak systolic velocity (PSV) both decreased significantly after changing to the supine posture (assessed after 15 min of rest) from the left lateral decubitus posture (also assessed after 15 min of rest) (Khatib et al., 2014). The authors did not calculate the change in the cerebroplacental ratio, which we calculated for reference: the cerebroplacental ratio decreased from 2.0 (54th percentile) in the left lateral decubitus posture to 1.73 (30th percentile) in the supine posture. Silva *et al.* (*N* = 20) did a similar study at the same gestational age range (Silva et al., 2017). They reported that the MCA PI in the left lateral posture (mean 1.7) was significantly reduced at 5 minutes after assuming the supine posture (mean 1.4). The findings of these studies suggest that the maternal supine posture may present a physiological stress sufficient to induce fetal adaptation via cerebral redistribution (also known as the “fetal brain sparing phenomenon”). In addition to cerebral hemodynamics, maternal posture may also affect fetal cerebral oxygenation. Aldrich et al. (*N* = 14) found a significant decrease in the mean concentration of cerebral oxyhemoglobin and mean cerebral oxygen saturation in the fetus in the maternal supine posture compared to left lateral in term pregnancies in uncomplicated labor with effective epidural analgesia (Aldrich et al., 1995). That said, there was no significant difference in fetal mean concentration of deoxyhemoglobin and fetal cerebral blood volume.

### Fetal behavioral state

Recently, Stone et al*.*, in a first-of-its-kind study, presented good evidence that maternal posture affects fetal behavioral state (FBS), fetal heart rate variability (fHRV), and mean fetal heart rate in a manner where the fetus resorts to a protective, quiescent, low oxygen consuming state when the mother assumes the supine posture. This phenomenon is likely due to reduced uteroplacental perfusion and subsequent mild hypoxic stress induced by this posture (Stone et al., 2017). In the supine and right lateral postures, there is more FBS 1F (quiet sleep, fetal quiescence) compared to the left lateral posture. When in the supine and semi-recumbent postures, there is less fHRV, less FBS 4F (active awake), and an FBS state change is more likely to occur when compared to lateral postures (Stone et al., 2017).

### Fetal growth

While fetal growth is less easily observed than measuring the FHR or other fetal blood flow indices, Hohmann and Künzel hypothesized that birthweight is related to the maternal mean arterial blood pressure (MAP) response to orthostatic stress in late pregnancy, namely, that pregnancies in which MAP falls upon standing upright (“orthostatic hypotension”) would give birth to smaller infants (Hohmann and Künzel, 1991). Indeed, they found a linear relationship between the change in MAP in response to orthostatic stress in late pregnancy and birthweight. In other words, birthweight was directly related to the magnitude and direction of the change in MAP with standing in late pregnancy. We return to the impact of maternal posture on fetal growth in the Activities section below.

## Maternal activities

In this section, we report on studies looking at posture during various maternal activities and their potential impact on fetal physiology. We divided this section into publications we found related to maternal sleep, occupation, exercise, driving and transportation, and sexual intercourse.

### Sleep

Approximately one-third of pregnancy is spent asleep. As such, sleep during pregnancy is a burgeoning area of research (Kember et al., 2023). We found that studies of maternal sleeping posture centered around three outcomes—placenta site, fetal growth, and late stillbirth—which we review in detail here. Other outcomes, such as the association between maternal sleeping posture on fetal occiput positioning *in utero* (Matsuo et al., 2007), had only one study and are not discussed further.

#### Placenta site

The specific location of the placenta within the uterus is an important clinical issue and is a defining feature of numerous placental disorders, for example, lateral placentation (Porto et al., 2020), placenta previa (Jain et al., 2020), and placenta accreta spectrum (PAS) disorders (Jauniaux et al., 2021). Despite this, however, we only found two studies that specifically looked at whether placenta site might be related to maternal posture. In one study of 500 pregnancies, Koken et al. found that supine sleeping posture was significantly associated with anterior placenta (odds ratio [OR]: 11.568, 95% confidence interval [CI]: 2.720–49.193) and prone sleeping posture was associated with posterior placenta (OR: 15.449, 95% CI: 2.151–52.978) (Koken et al., 2014). Participants who favored sleeping in the right lateral posture were more likely to have lateral placentation, while those who favored sleeping in the left lateral posture were more likely to have fundal placentation. In the other study, which included 1,500 pregnancies, Magann et al*.* found that those who usually slept supine at the time of conception and implantation were significantly more likely to have a high or fundal placental location compared with those who usually slept in the prone posture (Magann et al., 2002). In addition, those who slept exclusively on their right side early in pregnancy were significantly more likely to have a right-sided placental location compared with those who slept exclusively on their left side.

In summary, sleeping position early in gestation may influence the site of placental implantation. The mechanism may not be due to gravitational force alone and, instead, may also be related to the effect of maternal posture on uterine perfusion and oxygenation, which are hypothesized to influence placental implantation (Koken et al., 2014). Data are lacking, however, and these studies are limited by recall bias, inaccuracy of subjective reporting of sleeping position, and by not accounting for either uterine version (anteversion vs retroversion) nor flexion (anteflexion vs. retroflexion).

#### Fetal growth

It is well known that maternal BP is lower when asleep than when awake, and this applies across all trimesters of pregnancy (Siamopoulos et al., 1996). Given that maternal posture also affects BP, the last decade has seen several investigators reporting on the impact of maternal sleeping posture on pregnancy outcomes, particularly fetal growth and late stillbirth, which we discuss here.

In 2013, Owusu et al. first reported that low birth weight mediated the relationship between maternal supine sleeping posture after 28 weeks gestation and late stillbirth (Owusu et al., 2013). Participants who reported supine sleep in the third trimester were more likely to give birth to low birth weight (<2500g) infants, and these low birth weight infants were more likely to be stillborn. In 2019, Anderson *et al.* completed a secondary analysis [four case-control studies (Stacey et al., 2011; Gordon et al., 2015; McCowan et al., 2017; Heazell et al., 2018), *N* = 1760] of an individual patient data meta-analysis and showed that, compared to the left-lateral going-to-sleep posture, a supine going-to-sleep posture after 28 weeks gestation was associated with a lower birth weight (adjusted mean difference −144 g; 95% CI, −253 to −36 g) (Anderson et al., 2019). Contextualized, a 144 g reduction in birth weight is tantamount to about seven less days of fetal growth *in utero* (assuming an average fetal gain of 24 g per day at term) (de Jong et al., 1999). Furthermore, they found that the supine going-to-sleep posture after 28 weeks was associated with a more than triple odds of giving birth to a small-for-gestational age infant based on the INTERGROWTH-21 standard (adjusted odds ratio [aOR] 3.23, 95% CI 1.37–7.59) (Anderson et al., 2019). Since the supine going-to-sleep posture in the third trimester is known to correspond to an average of 48% of the total sleep time spent supine (whereas a non-supine going-to-sleep posture corresponds to an average of 23% of the total sleep time spent supine) (Wilson et al., 2022), these result suggest that supine sleeping posture negatively impacts fetal growth.

As for a causal link between maternal supine sleep and fetal growth, one potential mechanism could be the cumulative deficit in blood flow seen by the maternal-side of the placenta (intervillous space) during periods of supine sleep. A relatively simple calculation can be performed to estimate this by multiplying the columns of Table 1 to determine the volume of this cumulative deficit. The corresponding percentage of total blood flow to the intervillous space that this cumulative deficit represents is also given in Table 1. It is therefore intuitive to conclude that posture-dependent physiology may detrimentally affect fetal growth.

**TABLE 1:** Cumulative deficit in blood flow seen by the intervillous space for different percentages of the night spent sleeping supine throughout the third trimester, with corresponding percentage of total blood flow to the intervillous space that each cumulative deficit represents.

Proportion of night spent sleeping supine	Proportion uterine artery flow is reduced when supine^a^	Liters of blood per minute perfusing the uterus^b^	Proportion of uterine flow perfusing the placenta^c^	Number of minutes spent sleeping in third trimester^d^	Cumulative deficit	
					Liters	Percentage of total blood flow to intervillous space^e (%)^
0.27^f^	0.24	0.60	0.80	40,320	1,254	2.2
0.33^g^					1,532	2.6
0.48^h^					2,230	3.8

a There is a 24% reduction in uterine artery flow when supine in the third trimester. (Couper et al., 2021).

b In the third trimester, an average of 0.6 L of blood per minute perfuses the uterus. (Cunningham et al., 2023).

c Eighty percent of the uterine flow perfuses the placenta (while 20% perfuses the myometrium). (Cunningham et al., 2023).

d Assuming 8 hours per day are spent sleeping (or resting in bed) from 28 through 40 weeks gestation.

e Total blood flow to the intervillous space is calculated as: 0.60 L/min * 0.80 * 60 min/h * 24 h/day * 7 days/week * 12 weeks.

f The average amount of the night spent supine in the third trimester in studies where sleeping posture was confirmed by continuous overnight video recordings is 27%. (Maasilta et al., 2001; Wilson et al., 2011; OBrien and Warland, 2014; Warland and Dorrian, 2014; McIntyre et al., 2016; Warland et al., 2018a; Kember et al., 2018; Dunietz et al., 2020; Wilson et al., 2022).

g The average percent of the night spent supine in the third trimester if the waking-up posture in the morning was supine is 33%. (Wilson et al., 2022).

h The average percent of the night spent supine in the third trimester if the going-to-sleep posture was supine is 48%. (Wilson et al., 2022).

Sleeping posture in the third trimester is modifiable via “positional therapy” (PT) both in the laboratory and home settings (Warland et al., 2018a; Kember et al., 2018). Minimization of supine sleep using PT does not seem to affect sleep quality or quantity based on data from the gold-standard, polysomnography (Kember et al., 2018), and a level III ambulatory sleep device (Warland et al., 2018a). In pregnancies with obstructive sleep apnea, PT has been shown to improve overnight maternal respiratory parameters and the FHR tracing (Warland et al., 2018a). That said, a study by Lucchini et al*.* (*N* = 42) was unable to demonstrate an effect of maternal sleeping posture on the FHR. Major limitations of this study, however, were that the median percentage of time spent in the supine posture overnight was only 1.09% (IQR: 0.06%–5.05%) and sleeping position was not verified by video (Lucchini et al., 2020). Finally, it is worth noting that there has been one prospective study of PT in pregnancy. In a double-blind, sham-controlled, RCT of nightly PT used across the third trimester for minimization of supine sleep, Coleman et al. (*N* = 162) found a trend toward higher customized birth weight centile in the treatment group compared to the sham-treatment group (median 43% vs. 31%, *p* = 0.11) (Coleman et al., 2019). A reanalysis of this data using an updated maternal ethnicity coefficients showed similar results (median 42% vs. 28%, *p* = 0.06) (Coleman et al., 2024). A Bayesian analysis of this data indicates a high-to almost certain-probability that nightly maternal PT used throughout the third trimester will benefit fetal growth (Coleman et al., 2024).

#### Late stillbirth

High-quality evidence from an individual patient data meta-analysis by Cronin et al. in 2019 showed that, compared to the left-lateral going-to-sleep posture, a supine going-to-sleep posture after 28 weeks gestation was associated with more than double odds of stillbirth (aOR 2.63, 95% CI 1.72 to 4.04, five case-control studies (Stacey et al., 2011; Gordon et al., 2015; McCowan et al., 2017; Heazell et al., 2018; OBrien et al., 2019), *N* = 3,108) (Cronin et al., 2019). There was no significant interaction between the assessed indicators of fetal vulnerability and supine going-to-sleep position (Cronin et al., 2019). This ultimately means that supine going-to-sleep posture is independently associated with late stillbirth in the general pregnant population, regardless of body size, baby size, smoking, recreational drug use, pre-existing hypertension or diabetes, fetal movements, or term versus preterm gestation. As such, sleeping posture is important for all third-trimester pregnancies. Regarding sleeping posture prior to 28 weeks gestation, the best evidence, which includes a prospective study of self-reported and objectively measured sleeping posture from 22 through 30 weeks by Silver et al, (2019), indicates that it does not impact pregnancy outcomes. Indeed, if aortocaval compression via the gravid uterus play a role in the etiology of stillbirth, the risk of term (≥37 weeks) stillbirth from supine sleep should be greater than risk of preterm (<37 weeks) stillbirth from supine sleep due to increasing mass of the gravid uterus, increasing aortocaval compression. This is precisely what McCowan et al. concluded in their 2017 report (the aOR of supine sleep for term stillbirth was 10.26, and the aOR of supine sleep for preterm stillbirth was 3.12) (McCowan et al., 2017).

Maternal self-reported supine sleep time is known to underestimate objectively-determined supine sleep time by an absolute value of about 7% (Kember et al., 2018), which translates to a relative underestimate of about 46%. As noted in the previous section, we uncovered nine studies in which sleeping posture during the third trimester of pregnancy was objectively verified via overnight video recordings. Averaging the results of these studies, 27% of the night is spent supine during the third trimester (Maasilta et al., 2001; Wilson et al., 2011; OBrien and Warland, 2014; Warland and Dorrian, 2014; McIntyre et al., 2016; Warland et al., 2018a; Kember et al., 2018; Dunietz et al., 2020; Wilson et al., 2022). Data from Rådestad *et al.*, which included 583 stillbirths in Sweden from 2000 through 2014, indicate that only one-third of participants reported left lateral going-to-sleep posture and one-quarter recalled supine waking posture on the day their stillbirth was diagnosed. This may indicate that there is room for improvement in optimizing maternal sleeping posture (Rådestad et al., 2016). Indeed, in an online survey of Australian hospitals in 2018 (*N* = 83 hospital respondents), clinicians reported following best practice recommendations regarding advice on sleeping position less than 20% of the time (Andrews et al., 2020a). This, along with the aforementioned evidence, has informed the inclusion of maternal sleep posture into the Australian Government’s National Stillbirth Action and Implementation Plan (Australian Government and Department of Health, 2020), and the Centre of Research Excellence in Stillbirth and state health departments’ Safer Baby Bundle (Andrews et al., 2020b). In the UK, the National Institute for Healthcare and Excellence also recently analyzed this evidence and incorporated maternal sleeping posture recommendations into the Royal College of Obstetricians and Gynaecologists’ guideline for antenatal care (National Institute for Health and Care Excellence et al., 2021; Overview Antenatal care Guidance, 2022). More work remains to be done in other countries. A scoping review and metaanalysis by Warland *et al.* proposed maternal sleeping posture as one of four key areas (along with sleep-disordered breathing, sleep quality, and sleep duration) poised for investigation in view of reducing poor fetal outcomes, including stillbirth (Warland et al., 2018b). Finally, a recent systematic review by Rossiter *et al.* concluded that data regarding maternal sleeping posture in low- and middle-resource countries is sparse, suggesting a need for research in these diverse geographic areas (Rossiter et al., 2021).

### Occupational

As we have previously highlighted that approximately one-third of pregnancy is spent asleep, we must also acknowledge that approximately the same amount of time is spent working. However, compared to the volume of literature relating to maternal sleeping posture, our search uncovered a much greater volume pertaining to maternal posture at work. As such, we focus our discussion on metaanalyses and systematic reviews that have been conducted pertaining to this literature and have divided this into the impact on maternal posture on pregnancy outcomes 1) prior to fetal viability (i.e., prior to approximately 23 weeks gestation) and 2) after fetal viability.

#### Pregnancy outcomes prior to fetal viability

There has been long debate over the impact of maternal occupational posture on pregnancy outcomes prior to viability (Gold and Tomich, 1994). Research relating to spontaneous miscarriage (SM) tends to focus on prolonged occupational standing (Eskenazi et al., 1994; Gold and Tomich, 1994; Fenster et al., 1997; Senturia, 1997; Samaraweera and Abeysena, 2010). Studies of fertility outcomes (infertility, fecundability, prolonged time to pregnancy) have focused on the association with prolonged standing, prolonged sitting, sedentary behavior (Burdorf et al., 2011; Russo et al., 2018; Foucaut et al., 2019; Mena et al., 2020). A systematic review by Palmer et al. reported that standing posture at work is unlikely to be associated with SM (Palmer et al., 2013a). They postulate that small effects seen in the literature are likely due to chance, bias, or confounding, and larger, higher quality studies have lower estimates of risk compared to smaller, poorer quality studies. Another systematic review and metaanalysis by the same authors covering six studies from 1966 through 2012 reported that standing more than 6 h per day was associated with a small increase in risk of SM (pooled relative risk [RR] 1.16, 95% CI 1.01–1.32) (Bonde et al., 2013). When they restricted their analysis to the two highest quality studies, the RR of standing for SM was increased but crossed unity (pooled RR 1.23, 95% CI 0.83–1.96). They concluded that given limited evidence, advising against high levels of prolonged occupational standing may be prudent in pregnancy and that high-risk pregnancies may warrant additional and tailored counseling. Indeed, one of the two studies they deemed as “high-quality,” Fenster et al. reported that for pregnant women with a history of two or more SM’s, occupational standing for more than 7 h per day was associated with SM (OR 4.3, 95% CI 1.6–11.7), which was not seen in pregnancies with no history of SM but with the same level of standing exposure (Fenster et al., 1997). As such, we recommend that future studies of maternal occupational posture, SM, and other previable pregnancy outcomes stratify analyses for maternal history, including previous pregnancy loss.

#### Pregnancy outcomes after fetal viability

We found several studies investigating the associations and impact of maternal posture at work with pregnancy outcomes after fetal viability. Standing was the most common posture reported (Barnes et al., 1991; Eriksen et al., 1991; Hartikainen et al., 1994; Henriksen et al., 1995a; Henriksen et al., 1995b; Fortier et al., 1995; Koemeester et al., 1995; Luke et al., 1995; Spinillo et al., 1995; Cerón-Mireles et al., 1996; Spinillo et al., 1996; Hatch et al., 1997; Senturia, 1997; Lin et al., 1998; Newman et al., 2001; Ha et al., 2002; Saftlas et al., 2004; Saurel-Cubizolles et al., 2004; Magann et al., 2005; Pompeii et al., 2005; Croteau et al., 2006; Bonzini et al., 2009; Abeysena et al., 2010a; Abeysena et al., 2010b; Snijder et al., 2012; Agopian et al., 2017; Buen et al., 2020; Legesse et al., 2020), and some studies considered “demanding postures” (Croteau et al., 2007), stooping (Koemeester et al., 1995), trunk bending (Bonzini et al., 2009; Agopian et al., 2017), twisting (Agopian et al., 2017), squatting (Koemeester et al., 1995; Bonzini et al., 2009; Legesse et al., 2020), kneeling (Bonzini et al., 2009; Agopian et al., 2017), sitting (Koemeester et al., 1995; Spinillo et al., 1995; Saftlas et al., 2004; Agopian et al., 2017), and, while not necessarily maternal postures, keeping balance (Agopian et al., 2017) and occupational sedentary behavior (Hrubá et al., 1999; Saftlas et al., 2004). Other studies focused on specific occupations associated with various postures such as nurses (Koemeester et al., 1995; Luke et al., 1995), clerical workers (Spinillo et al., 1995), laboratory workers (Halliday-Bell et al., 2010), hairdressers (Halliday-Bell et al., 2009; Ronda et al., 2010), and cosmetologists (Halliday-Bell et al., 2009). The main outcomes of these studies were preterm birth (PTB) (Barnes et al., 1991; Henriksen et al., 1995b; Fortier et al., 1995; Luke et al., 1995; Cerón-Mireles et al., 1996; Newman et al., 2001; Saurel-Cubizolles et al., 2004; Magann et al., 2005; Pompeii et al., 2005; Arafa et al., 2007; Croteau et al., 2007; Bonzini et al., 2009; Halliday-Bell et al., 2009; Abeysena et al., 2010a; Halliday-Bell et al., 2010; Snijder et al., 2012; Buen et al., 2020) and indices of fetal growth (including fetal growth restriction (Spinillo et al., 1996), SGA (Hartikainen et al., 1994; Fortier et al., 1995; Cerón-Mireles et al., 1996; Pompeii et al., 2005; Croteau et al., 2006; Arafa et al., 2007; Bonzini et al., 2009; Halliday-Bell et al., 2009; Halliday-Bell et al., 2010; Snijder et al., 2012), birth weight [Barnes et al., 1991; Hartikainen et al., 1994; Henriksen et al., 1995a; Hatch et al., 1997; Senturia, 1997; Ha et al., 2002; Halliday-Bell et al., 2009; Abeysena et al., 2010b; Halliday-Bell et al., 2010; Ronda et al., 2010; Snijder et al., 2012; Legesse et al., 2020), head circumference (Bonzini et al., 2009; Snijder et al., 2012), and abdominal circumference (Bonzini et al., 2009)]. Other outcomes, however, included congenital malformations (Lin et al., 1998; Hrubá et al., 1999; Agopian et al., 2017), placental abnormalities (Hrubá et al., 1999), placental abruption (Eriksen et al., 1991), preeclampsia (Spinillo et al., 1995; Saftlas et al., 2004), gestational hypertension (GH) (Saftlas et al., 2004), gestational age at delivery (Koemeester et al., 1995; Senturia, 1997), and perinatal mortality (Senturia, 1997; Arafa et al., 2007; Halliday-Bell et al., 2009; Halliday-Bell et al., 2010). These numerous reports on occupational posture demonstrate occupational medicine’s commitment to maternal-fetal health and provide mature area of investigation ripe for meta-analytic studies, which we turn to now.

A meta-analysis by Mozurkewich et al*.* through August 1999 reported on 160,988 participants in 29 studies and found that prolonged occupational standing was associated with PTB (OR 1.26, 95% CI 1.13–1.40) (Mozurkewich et al., 2000). On the other hand, a systematic review by Palmer et al*.* reported that standing posture at work is unlikely to be associated with PTB, SGA, low birth weight, preeclampsia, and GH (Palmer et al., 2013a). They also report an updated meta-analysis of 86 studies up to the year 2011 wherein they concluded that the balance of evidence of maternal standing posture is against large effects (i.e., RR > 1.2) with best estimates pointing to modest or null effects for PTB and null effects for SGA (Bonzini et al., 2007; Palmer et al., 2013b). They report insufficient evidence relating occupational standing to preeclampsia and GH. Around the same time, van Beukering *et al.* reported a systematic review and meta-analysis, including 17 studies with low to moderate risk of bias from 1990 through 2012, and found that occupational standing for more than 3 hours per day was associated with PTB (OR 1.3, 95% CI 1.1–1.6) (van Beukering et al., 2014). Most recently, a metaanalysis by Cai *et al.* through March 2019 reported on 853,149 participants in 80 observational studies and found that prolonged occupational standing was associated with PTB (OR 1.11, 95% CI 1.02–1.22) and giving birth to an SGA infant (OR 1.17, 95% CI 1.01–1.35) (Cai et al., 2020). The unique contribution of their meta-analysis is a dose-response analysis that showed a 10% increase in odds of PTB in those who stand for more than 2.5 h per day at work compared to those who do not stand at work, suggesting that the ill-effects of exposure to upright posture is cumulative.

In summary, maternal occupational posture, specifically prolonged standing, likely results in small-to moderate-elevations in risks to the fetus. While much work has been done to delineate potential impacts of maternal occupational posture on pregnancy outcomes, we make a few recommendations for future research. First, researchers investigating PTB should delineate on the type (e.g., spontaneous, medically-indicated) and, if known, the etiology (e.g., prelabor premature rupture of membranes) of PTB. Second, as we found the standing posture to be most commonly reported, study protocols should strive to consider and report posture with more resolution than just standing and non-standing. Third, studies could report more detail on each posture—taking standing, for example, where reports could include the frequency of breaks to sit or lie down, activities while standing (e.g., bending, lifting, squatting, kneeling, twisting, turning, walking), and whether standing supported or unsupported. Fourth, the time spent in each posture should be precisely measured so dose-response relationships, if any, can be teased apart. Finally, investigators should also describe the type of work performed rather than relying on maternal occupational posture alone as a proxy for exposure. We did not find any occupational studies that used wearables and suggest that integration of wearables into such research may help accomplish several of these recommendations.

### Exercise

The landscape of research focusing on the impact of physical activity on fetal outcomes is vast. Historically, recommendations were made to reduce physical exercise during pregnancy out of fear there would be negative consequences such as pregnancy loss or reduced placental circulation (Barakat et al., 2008). It is now generally well accepted, however, that physical exercise during pregnancy is important for promoting maternal health. The impact of specific exercise postures during pregnancy on fetal outcomes remains controversial and understudied. This section narrows in on studies that evaluated the impact of maternal posture during exercise on fetal birth outcomes.

A systematic literature review by Schlüssel et al. evaluated 37 studies and focused on occupational physical activities, including housework, and leisure exercise, and their impact on both maternal and fetal outcomes (Schlüssel et al., 2008). Very few studies investigated the impact of physical activity during pregnancy on fetal development, though it appears that light and moderate physical activity is not a risk factor for poor fetal outcomes. There may be an association, however, between specific activities such as prolonged standing or climbing stairs and outcomes such as SM, low birth weight, and PTB. It was concluded that many gaps exist in this field and more research is needed to elucidate the trends more clearly and identify the safety of specific exercises (Schlüssel et al., 2008).

Due to the theoretical concern of supine positioning impacting maternal hemodynamics, the effect of supine exercise on fetal outcomes (such as low birth weight, SGA, and fetal demise) was reviewed systematically by Mottola *et al.* (Mottola et al., 2019) The review evaluated seven “low” to “very low” quality studies which ultimately concluded insufficient evidence exists to suggest whether maternal supine exercise during pregnancy is associated with adverse fetal birth outcomes (Mottola et al., 2019). Interestingly, a prospective study conducted by Polis, Gussman, and Kuo report the maternal and fetal effects of 26 yoga postures, each held for 2 min in 25 pregnancies in the late third trimester (35 weeks and 0 days through 37 weeks and 6 days) (Polis et al., 2015). Vital signs, pulse oximetry, uterine tocometry, and FHR were measured continuously through all postures and remained normal for all participants for the duration of the study. The participants did not experience any decreased fetal movement, contractions, leakage of fluid, or vaginal bleeding in the 24-h follow up. Even postures suspected to have a deleterious impact on the maternal or fetal physiology, including supine postures (Child’s Pose, Corpse Pose, Happy Baby Pose), did not cause any changes in normal monitoring. All postures were well tolerated and held longer than is done traditionally in yoga classes suggesting that yoga and many common supine yoga postures may be safe for the mother and fetus in the third trimester (Polis et al., 2015).

Avery *et al.* investigated changes in maternal HR, FHR, and maternal BP during moderate strength training conditioning exercises performed during two laboratory exercise sessions in 12 healthy pregnancies during the third trimester compared to non-pregnant controls (Avery et al., 1999). They found that the FHR baseline remained normal during and after maternal strength conditioning exercises. While in the sitting posture, no fetal bradycardia or FHR decelerations were observed during exercise and a very small number of decelerations were observed post-exercise. Moderate fetal bradycardia and FHR decelerations were observed occasionally while in the supine posture with 30° of upper body elevation before, during, and after exercise suggesting this posture may not be safe in the third trimester due to redistribution of blood flow during exercise. The increased frequency of FHR decelerations, however, was not statistically significantly compared to when the participants were in the sitting posture. The training may lead to increased fetal wakefulness as demonstrated by significant increases in frequency of FHR accelerations during double leg exercises in the seated posture (Avery et al., 1999). Overall, the results do not suggest that maternal or fetal health is significantly compromised during strength conditioning exercise in the third trimester, but the supine posture with 30° of upper body elevation might be best avoided to avoid potential fetal compromise.

Weng, Lee, and Chien found that reduced sitting time in late pregnancy had a negative effect on SGA (Weng et al., 2020). While this may suggest a protective effect of sitting, a reduction in sitting may also reflect increased participation in activities which negatively impact the fetus such as prolonged standing. Fazzi *et al.* conducted a systematic review to assess the impact of time spent in sedentary behaviors during pregnancy and how this may impact fetal outcomes (Fazzi et al., 2017). They found conflicting associations between sedentary behavior and birthweight, with some studies suggesting increased sedentary behavior is linked to larger newborn abdominal circumference and macrosomia, while other studies suggested lower birth weight was associated with increased sedentary behavior.

Overall, the great heterogeneity in exercises and outcomes as well as the volume and quality of studies limits the ability to draw conclusive advice around specific postures during exercise and the impact on the fetus. Higher quality methodology and increased investigation is needed to better elucidate the effect of various exercise postures and prolonged sitting and standing on fetal outcomes.

### Driving and transportation

We found few studies of maternal posture during driving and transportation and fetal physiology. In a study of pregnancies by Kromka-Szydek et al., 90% of participants reported sitting during travel in a car, bus, and tram, and 24%, 18%, and 8% of participants reported enhanced fetal movements in these modes of transportation, respectively (Kromka-Szydek et al., 2018). The investigators hypothesized that more intense fetal movements while riding in a car, in which sitting posture is standard, are a direct result of more exposure to vertical vibratory accelerations (transmitted through the seat and backrest) within the range of resonance frequencies of vital organs in the abdominal cavity (Kromka-Szydek et al., 2018). A study by Irannejad Parizi et al. looked at the forces applied to the gravid uterus and fetus (in head-down, occipito-anterior position) while riding in a passenger vehicle and experiencing sudden acceleration by passing over a speed-bump (Irannejad Parizi et al., 2021). Using their model, they recommend that a driver should not hit a standard speed-bump (0.5 m wide and 0.12 m tall) at 42 km/h or faster in order to avoid risk of fetal head injury. They advise hitting such speed-bumps under 25 km/h, and their model can be used to determine speed-bump dimensions that will avoid fetal injury at any desired vehicle speed.

### Sexual intercourse

Research shows that while the majority of pregnant patients believe that questions relating to coitus during pregnancy are important and should be discussed with their healthcare provider, these questions are only discussed with a minority (22%–29%) of patients (Bartellas et al., 2000; Uwapusitanon and Choobun, 2004), and a significant proportion of patients feel uncomfortable bringing up the topic themselves (Bartellas et al., 2000). In early pregnancy, heterosexual patients’ concerns center around coitus causing SM (Uwapusitanon and Choobun, 2004) and then give way to increasing concerns about coitus causing PTB and preterm prelabor rupture of membranes (PPROM) (Bartellas et al., 2000). With regard to maternal posture during sexual intercourse in pregnancy and its potential impact on fetal physiology, we found two reports. While Bartellas et al. did not report on fetal outcomes they did find a significant reduction in the reporting of “man on top” posture as gestation advances (Bartellas et al., 2000). These results provide context for a study by Ekwo et al. where only the missionary position was significantly associated with PPROM (OR 2.40, 95% CI 1.16–4.97) and PTB without PPROM (OR 1.82, 95% CI 1.02–3.25). Furthermore, no posture was found to be associated with term prelabor rupture of membranes. While these data are limited, they indicate that clinicians should discuss coitus with their antenatal patients and consider recommending alternatives to maternal supine posture (inferencing military position) as pregnancy progresses.

### Breastfeeding

In terms of maternal hand positions for introducing the breast to the infant, two that have been studied are palmar grasp and scissor grasp, and these two hand positions are no different when evaluated relative to infant milk intake (Altuntaş and Ünsal, 2019). As for maternal posture during breastfeeding, lactation experts suggest that maternal posture is an important component of breastfeeding education, reducing breastfeeding problems, and enhancing long-term breastfeeding (Henderson et al., 2001; Mbada et al., 2013). A recent meta-analysis, however, has demonstrated that the laid-back posture is beneficial for mitigating lactation-related nipple problems and is conducive to correct latching of the infant (Wang et al., 2021). An RCT of laid-back versus side-lying posture for breastfeeding following cesarean section found no statistically significant differences in breastfeeding outcomes related to the infant, but participants were more satisfied with the side-lying posture (Puapornpong et al., 2017), which may be related to experiencing less fatigue in this posture (Milligan et al., 1996).

## Medical procedures

Maternal posture during medical procedures may impact fetal physiology. See Supplementary material Data Sheet 2—Material Posture During Medical Procedures where we have organized our findings into fertility procedures, clinical growth assessment, imaging, external cephalic version, surgical procedures, advanced cardiovascular life support, and advanced trauma life support.

## Labor and birth

Upright postures during labor and birth have been documented for several millennia. In the last two centuries in the West, however, upright birthing postures have been largely eclipsed by supine postures, which is likely due to increasing use of epidural anesthesia (Dundes, 1987). While recent biomechanical studies indicate clear advantages of upright postures over non-upright postures vis à vis optimized pelvic dimensions for labor and birth (Reitter et al., 2014; Hemmerich et al., 2018; Desseauve et al., 2019; Hemmerich et al., 2019; Zhang et al., 2020), results of clinical studies should have the final say. We found numerous original research articles that considered maternal posture during labor and birth in relation to fetal physiology, including fetal oxygen saturation (Carbonne et al., 1996; Simpson and James, 2005), shoulder dystocia (Bruner et al., 1998; Buhimschi et al., 2001; Poggi et al., 2004; Nasir et al., 2007; Maheux-Lacroix et al., 2013; Zhang et al., 2017), malpresentation (Gizzo et al., 2014; Doyle et al., 2019), umbilical cord blood pH (Scholz et al., 2001; Bodner-Adler et al., 2003; Bick et al., 2017), Apgars (Waldenström and Gottvall, 1991; Bomfim-Hyppólito, 1998; Bodner-Adler et al., 2003; Maheux-Lacroix et al., 2013; Bick et al., 2017; Moraloglu et al., 2017; Zhang et al., 2017; Doyle et al., 2019), need for resuscitation at birth (Bick et al., 2017), and neonatal intensive care unit (NICU) admission (Waldenström and Gottvall, 1991; Bick et al., 2017; Moraloglu et al., 2017). In this discussion, however, we only consider fetal outcomes that were included in previous systematic reviews and meta-analyses of this topic.

Furthermore, we found several studies that included fetal outcomes in the investigation of maternal postural effects during administration of epidural (Preston et al., 1993; Eberle et al., 1998; Beilin et al., 2000; de la Chapelle et al., 2006), spinal (Køhler et al., 2002; Coppejans et al., 2006; Hwang et al., 2012; Zasa et al., 2015), or combined spinal-epidural anesthesia (Xu et al., 2017; Zhou et al., 2008; al-Mufti et al., 1997) either in labor or prior to an elective cesarean section (CS). These studies mainly focused on FHR (Beilin et al., 2000; Eberle et al., 1998; Preston et al., 1993; al-Mufti et al., 1997), Apgar scores (Coppejans et al., 2006; Zhou et al., 2008; Hwang et al., 2012; Zasa et al., 2015; Xu et al., 2017), and umbilical cord blood pH (Coppejans et al., 2006; Zhou et al., 2008; Zasa et al., 2015; Xu et al., 2017). We do not present these results here as no systematic reviews or meta-analyses were found in our search.

### First stage of labor

A 2013 meta-analysis by Lawrence et al. has shown a reduction in NICU admissions (RR 0.20, 95% CI 0.04–0.89) in patients without epidural anesthesia who alternated between the upright (walking, sitting, standing and kneeling) and recumbent (supine, semi‐recumbent, and lateral) postures in the first stage of labor compared to those who were in the recumbent posture only (Lawrence et al., 2013). This conclusion, however, was based on the result of only one RCT of 200 participants. For patients with an epidural, there was no difference in fetal outcomes between the same postural groups (upright and recumbent versus recumbent only) (Lawrence et al., 2013). In an older meta-analysis (2004) of patients with an epidural in the first stage of labor, Roberts *et al.*‘s analyzed five trials (*N* = 1,161) and concluded that there was no significant difference in Apgar scores between patients who ambulated compared to those who remained recumbent (Roberts et al., 2004).

### Second stage of labor

A 2017 updated meta-analysis by Gupta *et al.* has shown a reduction in abnormal FHR patterns (RR 0.46, 95% CI 0.22–0.93, 2 trials, *N* = 617) in any upright posture in the second stage in patients without an epidural compared to supine postures regardless of gravidity (Gupta et al., 2017). There was no difference in the number of infants admitted to NICU (RR 0.79, 95% CI 0.51–1.21, 4 trials, *N* = 2,565 infants), but the quality of evidence was low. In patients with an epidural, Walker et al.’s updated meta-analysis in 2018 reported fewer infants born with low cord pH in the upright group (RR 0.43, 95% CI 0.20–0.90, 2 trials, 3,159 infants, moderate-quality evidence) but did not identify any clear differences in abnormal FHR patterns requiring intervention (RR 1.69, 95% CI 0.32–8.84, one trial, N = 107), or admission to NICU (RR 0.54, 95% CI 0.02–12.73, one trial, *N* = 66 infants) (Kibuka and Thornton, 2017; Walker et al., 2018). Note, however, that the quality of evidence for the latter two outcomes was low, with more research required, particularly for additional fetal outcomes (e.g., Apgar scores, ventilatory requirements, perinatal death). An earlier meta-analysis (2005) of two trials (*N* = 281) by Roberts et al. reported that data were insufficient pertaining to infant outcomes and the possible effect of maternal posture (upright vs recumbent) in the second stage of labor with an epidural (Roberts et al., 2005). A 2020 meta-analysis by Dokmak et al. including seven RCT’s (*N* = 1,219) reported no difference in fetal outcomes for the maternal squatting posture (compared to supine) during the second stage of labor (Dokmak et al., 2020). They were unable to stratify for use of epidural anesthesia.

In summary, for patients without an epidural, upright postures in the first and second stage may confer fetal benefit, whereas data and postures are limited for patients with epidurals and further investigation is required. Note that the bulk of the evidence is regarding upright posture versus supine posture—few trials and no meta-analyses looked at supine versus a lateral, side-lying position in patients with epidural anesthesia, which may be beneficial since the majority of patients, in our region and demographic, opt for epidural anesthesia, which frequently limits posture options.

#### Fetal head malposition

There is debate as to whether maternal posture may affect fetal head position. A number of maternal postural techniques have been put forward to promote spontaneous rotation of the fetal head to occipito-anterior (OA) positions in order to increase successful vaginal delivery (Kariminia et al., 2004; Stremler et al., 2005; Gizzo et al., 2014; Guittier et al., 2016; Le Ray et al., 2016). In 2007, Hunter et al.‘s meta-analysis of three trials (*N* = 2,794) concluded that use of hands-and-knees posture for 10 minutes twice daily in late pregnancy cannot be recommended as an intervention to correct occipito-posterior (OP) fetal head position (Hunter et al., 2007). The use of hands-and-knees posture in labor, however, was associated with decreased back pain in patients with OP fetal head position. Another meta-analysis of five studies in 2007 by Ridley *et al.* concluded that in order to enhance rotation of the fetal head from OP to OA, the Sims’ posture on the same side as the fetal spine is recommended (Ridley, 2007). Finally, in 2021, Levy *et al.*’s meta-analysis of five studies (*N* = 1,727 hands-and-knees posture vs. *N* = 1,641 controls) also concluded that the hands-and-knees posture does not increase the rate of OA fetal head presentation at birth (Levy et al., 2021). Further studies are needed to truly determine if maternal posture influences on fetal head position.

#### Waterbirth

An object being acted on by the force of gravity has an additional force acting on it when placed in water: buoyant force. In a retrospective review of birth records (*N* = 6,144) over twelve-and-a-half years in a birthing center in Australia, Dahlen *et al.* reported that infants born in maternal semi-recumbent postures had a significantly greater incidence of 5-min Apgar score <7 (OR 4.61, 95% CI 1.29–16.52) compared to those born via waterbirth (Dahlen et al., 2013). However, a 2016 systematic review and metaanalysis by Taylor et al*.* of 29 studies found no significant difference in Apgar scores, cord gasses, infection rates, NICU admission, or neonatal mortality between infants born via water birth compared to those not born by waterbirth (maternal posture not reported).

### Third stage of labor

We found one meta-analysis (2010) on the influence of gravity (not maternal posture) on the newborn during the third stage of labor (the time from birth of the infant until delivery of the placenta) (Airey et al., 2010). However, no studies met the author’s inclusion criteria, and they concluded that large RCT’s are required in order to determine the effect of gravity on placental transfusion and fetal outcomes during vaginal birth and CS birth. A later non-inferiority RCT (2014) by Vain et al. concluded that the volume of placental transfusion is not affected by gravity (i.e., the position of the neonate prior to cord clamping) and that the neonate may be safely held on the mother’s abdomen or chest immediately after birth.

## Discussion

**FIGURE 2:**
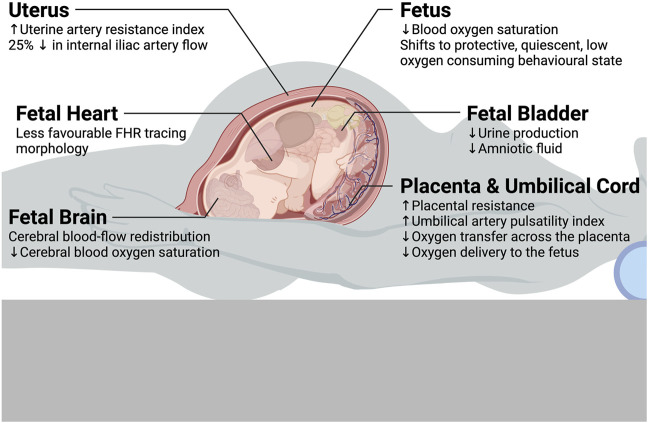
Summary diagram of changes in uterine, placental, umbilical cord, and fetal physiology observed in the maternal supine posture compared to lateral postures.

Regarding the potential impact that maternal posture has on fetal physiology, the supine posture emerges as a common culprit for potentially detrimental effects, with the exception of the upright posture in OUS (Schneider et al., 1984; Schneider and Deckardt, 1991; Schneider et al., 1993). See Figure 2 for a summary diagram of changes in uterine (Park and Hidaka, 1991; Ryo et al., 1996; Jeffreys et al., 2006; Couper et al., 2021), placental, (van Katwijk and Wladimiroff, 1991; Nakai et al., 1998; Khatib et al., 2014; Zun et al., 2017), umbilical cord (Abaci et al., 2020; Couper et al., 2021), and fetal (Aldrich et al., 1995; Cito et al., 2005; Simpson and James, 2005; Aluş et al., 2007; Tamás et al., 2007; Ulker et al., 2012a; Ulker et al., 2012b; Khatib et al., 2014; Silva et al., 2017; Stone et al., 2017; Couper et al., 2021; Ibrahim et al., 2021) physiology observed in the maternal supine posture compared to lateral postures.

The magnitude of these effects tend to increase with increasing gestation, which implicates aortocaval compression by the gravid uterus when supine is a primary mechanism underlying many of these observations. Given this, with the exception of cardiopulmonary resuscitation during advanced trauma and resuscitation (where left uterine displacement should be supplied by a second rescuer, see Supplementary material Data Sheet 2) (Jeejeebhoy et al., 2015; Panchal et al., 2020; Enomoto et al., 2022), the supine posture should be avoided in most medical procedures and maternal activities in late pregnancy. Practically, some investigators have suggested that Doppler velocimetry in the maternal supine posture or during orthostatic stress could be used as a “stress test” for the fetus (Schneider and Deckardt, 1991; Ryo et al., 1998). Others seeking clinical implications of maternal supine posture have described, for example, SHS resulting from being in the supine posture on the order of minutes. Further still, other investigators have looked at longer exposures to supine posture and found that the supine going-to-sleep posture is associated with SGA and late stillbirth (Anderson et al., 2019; Cronin et al., 2019). As such, some guidelines have adopted sleeping posture advice with regard to reducing stillbirth risk (Australian Government and Department of Health, 2020; Andrews et al., 2020b; National Institute for Health and Care Excellence et al., 2021; Overview Antenatal care Guidance, 2022). Likewise, in the case of OUS and the potential ill-effects of upright posture (Schneider and Deckardt, 1991), researchers have worked out that prolonged occupational standing is associated with an increased risk of SA (Fenster et al., 1997; Bonde et al., 2013), SGA (Palmer et al., 2013b; Cai et al., 2020), and PTB (Mozurkewich et al., 2000; Palmer et al., 2013b; van Beukering et al., 2014; Cai et al., 2020).

Conversely, this review has also found that maternal posture does not appear to impact other areas of pregnancy physiology. For example, during fertility treatments, posture immediately following embryo transfer (remaining supine or standing upright) does not seem to have an effect on fertility outcomes (see Supplementary material Data Sheet 2) (Abou-Setta et al., 2014; Craciunas and Tsampras, 2016; Cozzolino et al., 2019). Furthermore, the impact of maternal posture on some pregnancy physiology is debated, for example, during intrauterine insemination (see Supplementary material Data Sheet 2) (Saleh et al., 2000; Custers et al., 2009; van Rijswijk et al., 2017). For some of these controversies and reports of null findings, the quantity and quality of evidence is low. As such, the question becomes whether we have truly found evidence of absence of an effect or are dealing with absence of evidence of an effect. If the latter, which is often the case, more work remains to be done.

Throughout this review, we have highlighted several knowledge gaps, and note several additional areas warranting further study. First, dominant maternal sleeping posture around the time of conception may impact the placenta site (Magann et al., 2002; Koken et al., 2014). The mechanism may be due to the impact of maternal sleeping posture on uterine blood flow rather than the direct effect of maternal sleeping posture and gravity on the embryo itself. As such, we highlight this gap as an area which needs further investigation due to the potential major impact this could have at a population level, for example, on the incidence of PAS if an anterior placenta site could be avoided in pregnancies with prior CS. Second, we found a dearth of studies relating to maternal prone posture (Nakai et al., 1998; Oliveira et al., 2017; Dennis et al., 2018b), likely due to safety concerns. We recommend that more work be done to safely support the pregnant body while prone so that more research can be completed on this potentially therapeutic posture and close this gap. Third, given that the CS is the most common surgical procedure in the world (Jauniaux et al., 2016), there are still large knowledge gaps (Wilkinson and Enkin, 2007; Cluver et al., 2013), and we contend that more research into maternal posture during CS is needed, with separate analyses of elective CS and emergency CS. In passing, we note that the last meta-analysis on this topic was over a decade ago (Cluver et al., 2013). Finally, while sleep and work share roughly equal proportions of the 24 h day, we found that the amount of published studies of maternal sleeping posture is relatively small in comparison to those of maternal occupational posture. This gap should be addressed through more research into maternal sleeping posture, especially prospective, interventional trials. This is an exciting but understudied area as the only such trial to date indicates that avoidance of maternal supine sleep is highly likely to yield a fetal growth benefit (Coleman et al., 2024).

Within the text, to aid flow and context, we also made numerous recommendations for future research. We now present some additional recommendations to this end. First, it must be stated that none of the studies of placental, cord, or fetal physiology that we uncovered in our literature review accounted for the potential confounding effect of placental position. From a gravitational perspective, it is likely that the placental position is relevant after 26 weeks when the specific gravity is such that the fetus’ apparent weight is 0.6–0.8 of its actual weight (Sekulić et al., 2005) and, for example, a fetus with a posterior placenta would be resting on top of its placenta when its mother is lying supine. Second, we suggest that future research pertaining to maternal postural-dependent FHR changes should include beat-to-beat fHRV, which can yield insight into fetal autonomic tone that is not otherwise possible by analyzing FHR tracing morphology alone (Shuffrey et al., 2019; Kupka et al., 2020). On a related note, we encourage other innovative approaches like that of Stone *et al.* and their investigation of the FBS with maternal postural changes (Stone et al., 2017). Finally, studies of maternal lateral tilt should pay close attention to how the tilt is achieved—rolling to 15° left lateral tilt from the left lateral posture produces a completely different maternal hemodynamic state than rolling to the same position from the supine posture (Kundra et al., 2012). Tilt measurements should be defined and standardized because the angle of tilt achieved by the angle of a surface (e.g., operating table) significantly underestimates the angle of lateral tilt of the patient’s pelvis (Kinsella and Harvey, 2012).

## Figures and Tables

**Table:** 

**AFI**	Amniotic fluid index
**AFV**	Amniotic fluid volume
**AOR**	Adjusted odds ratio
**BOLD**	Blood-oxygen-level-dependent
**BP**	Blood pressure
**CI**	Confidence interval
**CO**	Cardiac output
**CS**	Cesarean section
**CTG**	Cardiotocography
**ECV**	External cephalic version
**FBS**	Fetal behavioral state
**FHR**	Fetal heart rate
**fHRV**	Fetal heart rate variability
**FiO2**	Fraction of inspired oxygen
**GH**	Gestational hypertension
**HR**	Heart rate
**IVC**	Inferior vena cava
**MAP**	Mean arterial blood pressure
**MCA**	Middle cerebral artery
**MRI**	Magnetic resonance imaging
**NICU**	Neonatal intensive care unit
**NPV**	Negative predictive value
**OA**	Occipito-anterior
**OP**	Occipito-posterior
**OR**	Odds ratio
**OUS**	Orthostatic uterovascular syndrome
**PAS**	Placenta accreta spectrum
**PI**	Pulsatility index
**pO2**	Partial pressure of oxygen
**PPROM**	Preterm prelabor rupture of membranes
**PPV**	Positive predictive value
**PT**	Positional therapy
**PTB**	Preterm birth
**RCT**	Randomized controlled trial
**S/D**	Systolic/diastolic
**SGA**	Small-for-gestational-age
**SHS**	Supine hypotensive syndrome
**SM**	Spontaneous miscarriage
**RI**	Resistance index
**RR**	Relative risk
**UA**	Umbilical artery
**UtA**	Uterine artery

## Footnotes

1 Kember, A. J., Anderson, J. L., Gorazd, N. E., House, S. C., Kerr, K., Torres, P. A., et al. Maternal posture-physiology interactions in human pregnancy: a narrative review. Under review.
