# Supplementary material for: Impact of maternal posture on fetal physiology in human pregnancy: a narrative review
Source: Front Physiol. 2024 May 17;15:1394707. doi: 10.3389/fphys.2024.1394707 (PMC11140392; doi:10.3389/fphys.2024.1394707)
Supplement: Supplementary file 1 [file DataSheet2.docx]

Supplementary material 2 – Maternal Posture During Medical Procedures

Maternal posture during medical procedures may impact fetal physiology. Here, we have organized our findings into fertility procedures, clinical growth assessment, imaging, external cephalic version, surgical procedures, advanced cardiovascular life support, and advanced trauma life support.

# Fertility

Most of the literature we found relating to maternal posture in the periconceptional period was related to fertility procedures. For oocyte retrieval, we found one case report reporting use of the lateral recumbent posture in a patient with severe thigh adduction contracture preventing the standard supine lithotomy posture.[(1)](https://www.zotero.org/google-docs/?GMwzeE)

Traditionally, supine posture and bed rest following embryo transfer (ET) has been recommended as a way to prevent embryo expulsion by gravity.[(2)](https://www.zotero.org/google-docs/?IT94PP) However, this has been challenged by more recent evidence in a Cochrane Review by Abou-Setta *et al.*, which did not support remaining in the supine posture following ET for any particular length of time.[(3)](https://www.zotero.org/google-docs/?jB7rPg) A systematic review and meta-analysis by Craciunas and Tsampras in 2016 including four randomized controlled trials (RCT) and 757 participants showed that bed rest following ET did not improve clinical pregnancy rate (PR) and live birth rate but reduced the implantation rate (IR).[(4)](https://www.zotero.org/google-docs/?B45mK7) This was corroborated by Cozzolino *et al*. in a 2019 systematic review and metaanalysis of five RCTs and 1,002 women, which found no difference in clinical pregnancy, live birth rate, ongoing PR, miscarriage rate, multiple pregnancy rate, or IR with immediate mobilization follow ET in comparison to bed rest.[(5)](https://www.zotero.org/google-docs/?lHLt80) It is unclear whether these meta-analyses accounted for uterine anteversion or retroversion. Agarwal *et al.* reported no difference in clinical PR after ET between anteverted and retroverted uteri,[(6)](https://www.zotero.org/google-docs/?YGtTFP) but they did not account for maternal posture. It is thought that most retroverted uteri convert to an anteverted position during the ET procedure anyways, even with a full bladder.[(7)](https://www.zotero.org/google-docs/?En554o) We highlight uterine version because one study we found by Knutzen *et al.* implies that there is an interaction between uterine version and patient posture immediately following ET vis à vis optimization of PR as follows: 1) patients with an anteverted uterus should remain in the knee-chest posture, and 2) patients with a retroverted or axial uterus should remain supine.[(8)](https://www.zotero.org/google-docs/?1CQFJc)

The “embryo flash position” refers to the air bubbles seen at the time of ultrasound-guided ET. These air bubbles are thought to represent the position of the embryo as the embryo cannot be directly visualized by ultrasound. Following ET, the majority, but not all, embryo flashes undergo significant migration towards the fundus.[(9)](https://www.zotero.org/google-docs/?MoeSi3) The location of the embryo flash and the direction of its movement at 60 min, but not at 1 or 5 min after transfer, is associated with clinical outcome (higher IR and PR with location <15 mm from fundus), which challenges the traditional notion that the exact position of the embryo flash immediately following ET is related to clinical outcome.[(9)](https://www.zotero.org/google-docs/?ALyDtw) Another study corroborates this, which found that the position of the air bubbles after ET is related to PR with the highest PR when the air bubbles end up closer to the fundus.[(10)](https://www.zotero.org/google-docs/?AnuVfT) That said, the location of the embryo flash within the first 60 minutes of ET is an imperfect predictor of the embryo implantation site as it correlates this site in only half of cases.[(11)](https://www.zotero.org/google-docs/?RAVU7C) It seems that standing shortly after ET does not play a significant role in the final position of embryo flash and is unlikely to be a factor in determining the position of embryos transferred to the uterine cavity during treatment with *in vitro* fertilization.[(12,13)](https://www.zotero.org/google-docs/?6l1nLj) Taken together, these studies indicate that while the embryo undergoes significant migration following ET and the embryo implantation site is an important factor for PR, the embryo’s implantation site is influenced by factors other than gravity and maternal posture, such as uterine contractions,[(3,11)](https://www.zotero.org/google-docs/?ooOEbN) deposition site, and negative pressure generated by removal of the transfer catheter.[(3)](https://www.zotero.org/google-docs/?7cYE4c)

For intrauterine insemination (IUI), at least two RCT’s support the assertion that 10-15 minutes of supine immobilization following IUI is beneficial for PR, ongoing PR, and live birth rate.[(14,15)](https://www.zotero.org/google-docs/?Jzxk57) A later and larger RCT in the Netherlands, however, showed no positive effect of 15 minutes of supine immobilization following IUI on PR.[(16)](https://www.zotero.org/google-docs/?ZcMJvj)

# Clinical Growth Assessment

The symphysis-fundal height (SFH) measurement is a key assessment completed at each antenatal care appointment starting at 24 weeks’ gestation in singleton pregnancies.[(17)](https://www.zotero.org/google-docs/?IFhxp5) The SFH is a crude screening test that, if measuring small or large for gestational age, prompts the clinician to complete an ultrasound scan to assess fetal growth and wellbeing. In a study by Engstrom *et al.*, the SFH measurement was taken on pregnant participants in four different postures by four different clinicians.[(18)](https://www.zotero.org/google-docs/?qP1Z3E) Measurements were found to be significantly different in the four postures with the highest mean SFH in the supine posture and lowest mean SFH in the trunk elevated with knees flexed posture (1.69 cm lower than the supine SFH).[(18)](https://www.zotero.org/google-docs/?QizFAO) This study indicates the importance of clinicians standardizing their own technique for measuring SFH, with particular attention to maternal posture, in all antenatal visits.

# Imaging

## Cervix

Several papers over the last few decades have reported posture-dependent changes in cervical morphology, mainly through the use of ultrasound imaging, although this finding was not consistent and depended on the gestational age. For example, a study of patients with high risk pregnancies revealed that 14 of 16 participants where cervical length (CL) shortened by more than 33% when transitioning from supine to standing went on to have preterm birth (PTB).[(19)](https://www.zotero.org/google-docs/?Gb4moE) This was compared to only 1 of 25 in the group with smaller CL changes suggesting maternal postural related CL shortening could be used to identify patients at risk for PTB. Further work by Meijer-Hoogeveen *et al.* reported a study aimed at predicting PTB through the assessment of rapid CL shortening in the third trimester.[(20)](https://www.zotero.org/google-docs/?FOelxW) This longitudinal transvaginal ultrasound study reported minimal differences in CL between supine and upright postures, but the authors did conclude that CL measurements between 37 and 38 weeks gestation could be used to predict the onset of labor, which has doubtful clinical utility. Unfortunately, both the sensitivity and negative predictive value (NPV) of this approach was low, primarily due to inter-individual variations in CL. Similarly, Guzman *et al.* reported that CL shortening upon standing had a lower sensitivity for detecting a cervix that will undergo subsequent progressive shortening compared to transfundal pressure (33.3% vs. 83.3%) while specificity, positive predictive value (PPV), and negative predictive value (NPV) were similar.[(21)](https://www.zotero.org/google-docs/?JeCOaw) In another study by Meijer-Hoogeveen *et al.* the authors found that ultrasonographic changes in CL with maternal posture changes may more accurately predict successful vaginal delivery after induction of labor at term when compared with prediction via the Bishop score.[(22)](https://www.zotero.org/google-docs/?6WMjCC)

Other studies in twin and triplet pregnancies provide contradictory evidence with some data suggesting there was a difference in CL between recumbent and standing postures,[(23)](https://www.zotero.org/google-docs/?kkDWCv) while further reports found no significant differences.[(24)](https://www.zotero.org/google-docs/?bOcmhi) Part of this could be due to inter- and intraobserver differences when acquiring and reading ultrasound images of the cervix,[(25)](https://www.zotero.org/google-docs/?EFIVWU) although at least one report suggests upright posture improves the chances of detecting funneling of amniotic membranes through the cervix,[(25)](https://www.zotero.org/google-docs/?GzOGo7) indicating that the consistency of the cervix is soft. Another longitudinal study of 32 singleton, twin, and triplet pregnancies quantified CL via weekly ultrasound in standing and supine postures, with and without a Valsalva maneuver.[(26)](https://www.zotero.org/google-docs/?D7ruFZ) The results suggested that CL can differ significantly as pregnancy progresses and that the Valsalva maneuver temporarily may shorten the cervix due to the internal pressure it creates. Other body postures have also been studied, including a “hips up” maneuver where the patients’ hips are elevated by a cushion. This position helped improve transperineal imaging of the cervix when compromised by nearby bowel gas, but CL was not reported in this study.[(27)](https://www.zotero.org/google-docs/?LnVcna)

Taken together, these reports suggest maternal posture is a factor that should be taken into consideration when imaging and assessing the cervix of pregnant patients.

## Fetus

During fetal ultrasound in the second and third trimester, a study by Broome *et al.* (N=526) demonstrated that maternal upright seated posture improves visibility and assessment of the fetal spine in its posterior sagittal and axial projections for fetuses in the cephalic presentation when this exam is added to a routine examination in which visualization is incomplete.[(28)](https://www.zotero.org/google-docs/?cEfku1) Optimized coil coverage in the maternal supine posture during fetal magnetic resonance imaging (MRI) yields superior image quality compared with images collected in maternal non-supine postures.[(29)](https://www.zotero.org/google-docs/?OS1DUX) Due to fetal MRI acquisition taking up to 45 minutes, there is some apprehension to using the maternal supine posture due to concerns of precipitating supine hypotensive syndrome (SHS). This notion, however, has been challenged in a study by Kienzl *et al.* in which no participants imaged in the supine posture (N=56) experienced symptoms of SHS.[(30)](https://www.zotero.org/google-docs/?MqETTH) The median gestation in their study, however, was only 27 weeks and 4 days, and fetal physiology during the MRI’s was not assessed. As such, we suggest that more work is needed to elucidate the safety of the maternal supine posture during fetal MRI.

# External Cephalic Version

Cesarean section is the most common method of birth for breech fetuses. External cephalic version (ECV) is the manual external manipulation of the fetal position through the maternal abdomen in an attempt to turn the breech fetus such that it is head down (“cephalic version”) and, as such, increase the chance of vaginal delivery. Traditionally, maternal posture has been thought to affect fetal presentation, and various maternal postural techniques have been hypothesized to spontaneously promote cephalic version in non-cephalic fetuses.[(31,32)](https://www.zotero.org/google-docs/?O6hCeR) This convention has been challenged, however, in a meta-analysis by Hofmeyer and Kulier in 2012, which included six studies (N=417).[(31)](https://www.zotero.org/google-docs/?QweSuj) They investigated the effect of maternal posture (knee-chest posture, and a supine posture with the pelvis elevated with a wedge-shaped cushion) on cephalic version success in breech presentation and concluded that there is insufficient evidence to recommend maternal postural management for breech presentation. Further research is needed due to small sample sizes in the trials to date.

# Surgery

We found several reports of maternal posture during surgical procedures and report those with outcomes related to the fetus.

## Cerclage

There may be advantages to moving pregnant patients into a knee-chest posture when placing a cervical cerclage, or suture around the distal end of the cervix, as the amniotic membranes shift further away from the cervical canal and further into the uterus, reducing the risk of preterm prelabor rupture of membranes (PPROM) and PTB.[(33)](https://www.zotero.org/google-docs/?tOwgBr) In our institution (Mount Sinai Hospital, Toronto, Canada), we use the supine lithotomy posture with trendelenburg.

## Cesarean Section

Our search found that most reports of maternal posture during surgical procedures in pregnancy were related to cesarean section (CS). Given these numerous reports, we limit our discussion here to systematic reviews and meta-analyses.

The most recent systematic review of maternal posture during CS is by Cluver *et al.* in 2013 and included 11 studies.[(34)](https://www.zotero.org/google-docs/?r4SenX) They concluded that, despite limited evidence, left lateral tilt may be superior (less maternal hypotensive episodes) than right lateral tilt and that manual displacement of the uterus may be superior (less maternal hypotensive episodes, decreased fall in maternal mean systolic blood pressure) than left lateral tilt. As for fetal outcomes, they found no statistically significant differences in five-minute Apgars or cord blood pH between the various maternal postures evaluated. While they had planned to evaluate these outcomes in elective CS and emergency CS separately, they were unable to due to the small number of trials and sample sizes.

## Laparoscopy

The Society of American Gastrointestinal and Endoscopic Surgeons guideline for use of laparoscopy during pregnancy recommends placing gravid patients in the left lateral decubitus posture to minimize compression of the inferior vena cava (IVC) (moderate quality of evidence, strong recommendation)[(35)](https://www.zotero.org/google-docs/?YA1lND) and, presumably, resultant maternal-fetal effects. In special circumstances, however, such as laparoscopic left diaphragmatic hernia repair, the right lateral posture has been safely used.[(36)](https://www.zotero.org/google-docs/?0X7PT9)

## Neurosurgery

We found eight case reports,[(37–44)](https://www.zotero.org/google-docs/?pwvasS) one small case series [(45)](https://www.zotero.org/google-docs/?8rYeBi), and one systematic review [(46)](https://www.zotero.org/google-docs/?dqM4eG) reporting posture of pregnant patients during neurosurgery. Spinal surgery for herniated discs and burst fractures has been reported between 16 and 32 weeks, safely, in the left lateral, right lateral, prone (earlier gestation), and three-quarter prone postures (later gestation).[(38–40,44–46)](https://www.zotero.org/google-docs/?Fd5Byk) Posterior fossa craniotomy for various masses has been reported between 20 and 30 weeks, safely, in the prone, three-quarter prone, and sitting postures.[(41–43)](https://www.zotero.org/google-docs/?z6gylx)

## Urologic Surgery

For urolithiasis in early pregnancy (8 through 12 weeks), prone posture has been used successfully and safely in urgent ultrasound-guided percutaneous nephrolithotomy under general anesthesia.[(47)](https://www.zotero.org/google-docs/?NSw4A4) At later gestations, other authors used spinal anesthesia and completed the procedure in the supine posture at 16 weeks and 20 weeks and in the left lateral posture at 28 weeks.[(48)](https://www.zotero.org/google-docs/?b2LvPE)

# Advanced Cardiovascular Life Support & Advanced Trauma Life Support

The primary focus of Advanced Cardiovascular Life Support (ACLS) and Advanced Trauma Life Support (ATLS) in the setting of pregnancy is to save the life of the mother. As such, most of the literature relating to maternal posture during ACLS and ATLS does not report on fetal physiology or pathophysiology. That said, in our institution (Mount Sinai Hospital, Toronto, Canada), we have witnessed perimortem CS in which good-quality cardiopulmonary resuscitation (CPR) has ensured the birth of a healthy neonate despite maternal asystole prior to birth. Because fetal physiology is impacted by maternal posture, we report summary guidance relating to maternal posture during ACLS and ATLS for the sake of completeness. The American Heart Association scientific statement on cardiac arrest in pregnancy states that CPR and post-arrest care will be ineffective if aortocaval compression is not continuously relieved.[(49)](https://www.zotero.org/google-docs/?SLIzhH) To relieve aortocaval compression in the supine posture, left lateral tilt is not recommended because it has been demonstrated to compromise the quality of chest compressions with a 20% reduction in compression depth.[(50)](https://www.zotero.org/google-docs/?wSsczA) Instead, the American Heart Association’s 2020 guideline recommends that if the uterus is palpated at or above the umbilicus or at 20 weeks or more, continuous manual left uterine displacement should be performed on all pregnant patients who are in cardiac arrest.[(51)](https://www.zotero.org/google-docs/?TNa6dU) Manual left uterine displacement requires a second rescuer to cup the uterus pushing it leftward and upward off the IVC. In the setting of trauma, Canadian trauma guidelines for pregnancy recommend that after mid-pregnancy, manual left uterine displacement or left lateral tilt should be used.[(52)](https://www.zotero.org/google-docs/?2w07sQ) Note, however, that care should be taken to secure the spinal cord with a backboard when using left lateral tilt.

References

[1. Martins WP, Soares C a. M, Barbosa MWP, Yamaguti EMM, Ferriani RA. Oocyte retrieval using the lateral recumbent position. Ultrasound Obstet Gynecol Off J Int Soc Ultrasound Obstet Gynecol. 2016 Jul;48(1):126–7.](https://www.zotero.org/google-docs/?ti1Mjy)

[2. al-Shawaf T, Dave R, Harper J, Linehan D, Riley P, Craft I. Transfer of embryos into the uterus: how much do technical factors affect pregnancy rates? J Assist Reprod Genet. 1993 Jan;10(1):31–6.](https://www.zotero.org/google-docs/?ti1Mjy)

[3. Abou-Setta AM, Peters LR, D’Angelo A, Sallam HN, Hart RJ, Al-Inany HG. Post-embryo transfer interventions for assisted reproduction technology cycles. Cochrane Database Syst Rev. 2014 Aug 27;(8):CD006567.](https://www.zotero.org/google-docs/?ti1Mjy)

[4. Craciunas L, Tsampras N. Bed rest following embryo transfer might negatively affect the outcome of IVF/ICSI: a systematic review and meta-analysis. Hum Fertil Camb Engl. 2016 Apr;19(1):16–22.](https://www.zotero.org/google-docs/?ti1Mjy)

[5. Cozzolino M, Troiano G, Esencan E. Bed rest after an embryo transfer: a systematic review and meta-analysis. Arch Gynecol Obstet. 2019 Nov;300(5):1121–30.](https://www.zotero.org/google-docs/?ti1Mjy)

[6. Agarwal SK, Coe S, Buyalos RP. The influence of uterine position on pregnancy rates with in vitro fertilization-embryo transfer. J Assist Reprod Genet. 1994 Jul;11(6):323–4.](https://www.zotero.org/google-docs/?ti1Mjy)

[7. Henne MB, Milki AA. Uterine position at real embryo transfer compared with mock embryo transfer. Hum Reprod Oxf Engl. 2004 Mar;19(3):570–2.](https://www.zotero.org/google-docs/?ti1Mjy)

[8. Knutzen V, Stratton CJ, Sher G, McNamee PI, Huang TT, Soto-Albors C. Mock embryo transfer in early luteal phase, the cycle before in vitro fertilization and embryo transfer: a descriptive study. Fertil Steril. 1992 Jan;57(1):156–62.](https://www.zotero.org/google-docs/?ti1Mjy)

[9. Saravelos SH, Wong AWY, Chan CPS, Kong GWS, Cheung LP, Chung CHS, et al. Assessment of the embryo flash position and migration with 3D ultrasound within 60 min of embryo transfer. Hum Reprod Oxf Engl. 2016 Mar;31(3):591–6.](https://www.zotero.org/google-docs/?ti1Mjy)

[10. Lambers MJ, Dogan E, Lens JW, Schats R, Hompes PGA. The position of transferred air bubbles after embryo transfer is related to pregnancy rate. Fertil Steril. 2007 Jul;88(1):68–73.](https://www.zotero.org/google-docs/?ti1Mjy)

[11. Saravelos SH, Wong AWY, Chan CPS, Kong GWS, Li TC. How often does the embryo implant at the location to which it was transferred? Ultrasound Obstet Gynecol Off J Int Soc Ultrasound Obstet Gynecol. 2016 Jul;48(1):106–12.](https://www.zotero.org/google-docs/?ti1Mjy)

[12. Woolcott R, Stanger J. Ultrasound tracking of the movement of embryo-associated air bubbles on standing after transfer. Hum Reprod Oxf Engl. 1998 Aug;13(8):2107–9.](https://www.zotero.org/google-docs/?ti1Mjy)

[13. Lambers MJ, Lambalk CB, Schats R, Hompes PGA. Ultrasonographic evidence that bedrest after embryo transfer is useless. Gynecol Obstet Invest. 2009;68(2):122–6.](https://www.zotero.org/google-docs/?ti1Mjy)

[14. Saleh A, Tan SL, Biljan MM, Tulandi T. A randomized study of the effect of 10 minutes of bed rest after intrauterine insemination. Fertil Steril. 2000 Sep;74(3):509–11.](https://www.zotero.org/google-docs/?ti1Mjy)

[15. Custers IM, Flierman PA, Maas P, Cox T, Van Dessel TJHM, Gerards MH, et al. Immobilisation versus immediate mobilisation after intrauterine insemination: randomised controlled trial. BMJ. 2009 Oct 29;339:b4080.](https://www.zotero.org/google-docs/?ti1Mjy)

[16. van Rijswijk J, Caanen MR, Mijatovic V, Vergouw CG, van de Ven PM, Lambalk CB, et al. Immobilization or mobilization after IUI: an RCT. Hum Reprod Oxf Engl. 2017 Nov 1;32(11):2218–24.](https://www.zotero.org/google-docs/?ti1Mjy)

[17. Overview | Antenatal care | Guidance | NICE [Internet]. NICE; [cited 2022 Sep 2]. Available from: https://www.nice.org.uk/guidance/ng201](https://www.zotero.org/google-docs/?ti1Mjy)

[18. Engstrom JL, Piscioneri LA, Low LK, McShane H, McFarlin B. Fundal height measurement. Part 3--The effect of maternal position on fundal height measurements. J Nurse Midwifery. 1993 Feb;38(1):23–7.](https://www.zotero.org/google-docs/?ti1Mjy)

[19. Wong G, Levine D, Ludmir J. Maternal postural challenge as a functional test for cervical incompetence. J Ultrasound Med. 1997;16(3):169–75.](https://www.zotero.org/google-docs/?ti1Mjy)

[20. Meijer-Hoogeveen M, Van Holsbeke C, Van Der Tweel I, Stoutenbeek P, Visser GHA. Sonographic longitudinal cervical length measurements in nulliparous women at term: prediction of spontaneous onset of labor. Ultrasound Obstet Gynecol Off J Int Soc Ultrasound Obstet Gynecol. 2008 Oct;32(5):652–6.](https://www.zotero.org/google-docs/?ti1Mjy)

[21. Guzman ER, Pisatowski DM, Vintzileos AM, Benito CW, Hanley ML, Ananth CV. A comparison of ultrasonographically detected cervical changes in response to transfundal pressure, coughing, and standing in predicting cervical incompetence. Am J Obstet Gynecol. 1997 Sep;177(3):660–5.](https://www.zotero.org/google-docs/?ti1Mjy)

[22. Meijer-Hoogeveen M, Roos C, Arabin B, Stoutenbeek P, Visser GHA. Transvaginal ultrasound measurement of cervical length in the supine and upright positions versus Bishop score in predicting successful induction of labor at term. Ultrasound Obstet Gynecol Off J Int Soc Ultrasound Obstet Gynecol. 2009 Feb;33(2):213–20.](https://www.zotero.org/google-docs/?ti1Mjy)

[23. Arabin B, Aardenburg R, van Eyck J. Maternal position and ultrasonic cervical assessment in multiple pregnancy. Preliminary observations. J Reprod Med. 1997 Nov;42(11):719–24.](https://www.zotero.org/google-docs/?ti1Mjy)

[24. Bernáth T, Brizot ML, Liao AW, Cury L, Banduki Neto JD, Zugaib M. Effect of maternal position on cervical length measurement in twin pregnancies. Ultrasound Obstet Gynecol Off J Int Soc Ultrasound Obstet Gynecol. 2002 Sep;20(3):263–6.](https://www.zotero.org/google-docs/?ti1Mjy)

[25. Arabin B, Roos C, Kollen B, van Eyck J. Comparison of transvaginal sonography in recumbent and standing maternal positions to predict spontaneous preterm birth in singleton and twin pregnancies. Ultrasound Obstet Gynecol Off J Int Soc Ultrasound Obstet Gynecol. 2006 Apr;27(4):377–86.](https://www.zotero.org/google-docs/?ti1Mjy)

[26. Meath AJ, Ramsey PS, Mulholland TA, Rosenquist RG, Lesnick T, Ramin KD. Comparative longitudinal study of cervical length and induced shortening changes among singleton, twin, and triplet pregnancies. Am J Obstet Gynecol. 2005 May;192(5):1410–5.](https://www.zotero.org/google-docs/?ti1Mjy)

[27. Hertzberg BS, Kliewer MA, Baumeister LA, McNally PB, Fazekas CK. Optimizing transperineal sonographic imaging of the cervix: the hip elevation technique. J Ultrasound Med Off J Am Inst Ultrasound Med. 1994 Dec;13(12):933–6; quiz 1009–10.](https://www.zotero.org/google-docs/?ti1Mjy)

[28. Broome DR, Graichen MV, Omessi EM, Taber KH. Improved sonographic visualization of the fetal spine with the mother seated. AJR Am J Roentgenol. 1998 Mar;170(3):701–6.](https://www.zotero.org/google-docs/?ti1Mjy)

[29. Brugger PC, Stuhr F, Lindner C, Prayer D. [Fetal magnetic resonance imaging: methods and techniques]. Radiol. 2006 Feb;46(2):105–11.](https://www.zotero.org/google-docs/?ti1Mjy)

[30. Kienzl D, Berger-Kulemann V, Kasprian G, Brugger PC, Weber M, Bettelheim D, et al. Risk of inferior vena cava compression syndrome during fetal MRI in the supine position – a retrospective analysis. J Perinat Med. 2014 May 1;42(3):301–6.](https://www.zotero.org/google-docs/?ti1Mjy)

[31. Hofmeyr GJ, Kulier R. Cephalic version by postural management for breech presentation. Cochrane Database Syst Rev. 2012 Oct 17;10(10):CD000051.](https://www.zotero.org/google-docs/?ti1Mjy)

[32. Smith C, Crowther C, Wilkinson C, Pridmore B, Robinson J. Knee-chest postural management for breech at term: a randomized controlled trial. Birth Berkeley Calif. 1999 Jun;26(2):71–5.](https://www.zotero.org/google-docs/?ti1Mjy)

[33. Ogawa M, Sanada H, Tsuda A, Hirano H, Tanaka T. Modified cervical cerclage in pregnant women with advanced bulging membranes: knee-chest positioning. Acta Obstet Gynecol Scand. 1999 Oct;78(9):779–82.](https://www.zotero.org/google-docs/?ti1Mjy)

[34. Cluver C, Novikova N, Hofmeyr GJ, Hall DR. Maternal position during caesarean section for preventing maternal and neonatal complications. Cochrane Database Syst Rev. 2013 Mar 28;(3):CD007623.](https://www.zotero.org/google-docs/?ti1Mjy)

[35. Pearl J, Price R, Richardson W, Fanelli R, Society of American Gastrointestinal Endoscopic Surgeons. Guidelines for diagnosis, treatment, and use of laparoscopy for surgical problems during pregnancy. Surg Endosc. 2011 Nov;25(11):3479–92.](https://www.zotero.org/google-docs/?ti1Mjy)

[36. Julien F, Drolet S, Lévesque I, Bouchard A. The right lateral position for laparoscopic diaphragmatic hernia repair in pregnancy: technique and review of the literature. J Laparoendosc Adv Surg Tech A. 2011 Feb;21(1):67–70.](https://www.zotero.org/google-docs/?ti1Mjy)

[37. Patidar RK, Kaur M, Gosal JS, Garg M, Bhaskar S, Jha DK. Letter to the Editor Regarding “Positioning Issues of Spinal Surgery During Pregnancy.” World Neurosurg. 2020 Jul;139:694–5.](https://www.zotero.org/google-docs/?ti1Mjy)

[38. Hayakawa K, Mizutani J, Suzuki N, Haas C, Kondo A, Otsuka S, et al. Surgical Management of the Pregnant Patient With Lumbar Disc Herniation in the Latter Stage of the Second Trimester. Spine. 2017 Feb;42(3):E186–9.](https://www.zotero.org/google-docs/?ti1Mjy)

[39. Kovari VZ, Horvath L. Surgical management of cauda syndrome in third trimester of pregnancy focusing on spinal anesthesia and right lateral positioning during surgery as possible practices. Eur Spine J Off Publ Eur Spine Soc Eur Spinal Deform Soc Eur Sect Cerv Spine Res Soc. 2018 Jul;27(Suppl 3):483–8.](https://www.zotero.org/google-docs/?ti1Mjy)

[40. Sonawane DV, Nemade PS, Chandanwale A, Pawar E, Jagtap SA. Management of fracture dorso-lumbar spine in a pregnant female by surgery in prone position. Eur Spine J Off Publ Eur Spine Soc Eur Spinal Deform Soc Eur Sect Cerv Spine Res Soc. 2018 Jul;27(Suppl 3):270–5.](https://www.zotero.org/google-docs/?ti1Mjy)

[41. Casabella AM, Urakov TM, Basil G, Morcos JJ. Management of Foramen Magnum Meningioma During Pregnancy: Literature Review and Case Report. World Neurosurg. 2017 Jan;97:752.e15-752.e18.](https://www.zotero.org/google-docs/?ti1Mjy)

[42. Jacob J, Alexander A, Philip S, Thomas A. Prone position craniotomy in pregnancy without fetal heart rate monitoring. J Clin Anesth. 2016 Sep;33:119–22.](https://www.zotero.org/google-docs/?ti1Mjy)

[43. Giannini A, Bricchi M. Posterior fossa surgery in the sitting position in a pregnant patient with cerebellopontine angle meningioma. Br J Anaesth. 1999 Jun;82(6):941–4.](https://www.zotero.org/google-docs/?ti1Mjy)

[44. Schnake KJ, Scholz M, Marx A, Hoffmann R, Kandziora F. Anterior, thoracoscopic-assisted reduction and stabilization of a thoracic burst fracture (T8) in a pregnant woman. Eur Spine J Off Publ Eur Spine Soc Eur Spinal Deform Soc Eur Sect Cerv Spine Res Soc. 2011 Aug;20(8):1217–21.](https://www.zotero.org/google-docs/?ti1Mjy)

[45. Brown MD, Levi AD. Surgery for lumbar disc herniation during pregnancy. Spine. 2001 Feb 15;26(4):440–3.](https://www.zotero.org/google-docs/?ti1Mjy)

[46. Bongetta D, Versace A, De Pirro A, Gemma M, Bernardo L, Cetin I, et al. Positioning issues of spinal surgery during pregnancy. World Neurosurg. 2020 Jun;138:53–8.](https://www.zotero.org/google-docs/?ti1Mjy)

[47. Hosseini MM, Hassanpour A, Eslahi A, Malekmakan L. Percutaneous Nephrolithotomy During Early Pregnancy in Urgent Situations: Is It Feasible and Safe? Urol J. 2017 Nov 4;14(6):5034–7.](https://www.zotero.org/google-docs/?ti1Mjy)

[48. Basiri A, Nouralizadeh A, Kashi AH, Radfar MH, Nasiri MR, Zeinali M, et al. X-Ray Free Minimally Invasive Surgery for Urolithiasis in Pregnancy. Urol J. 2016 Mar 5;13(1):2496–501.](https://www.zotero.org/google-docs/?ti1Mjy)

[49. Jeejeebhoy FM, Zelop CM, Lipman S, Carvalho B, Joglar J, Mhyre JM, et al. Cardiac Arrest in Pregnancy: A Scientific Statement From the American Heart Association. Circulation. 2015 Nov 3;132(18):1747–73.](https://www.zotero.org/google-docs/?ti1Mjy)

[50. Enomoto N, Yamashita T, Furuta M, Tanaka H, Ng ESW, Matsunaga S, et al. Effect of maternal positioning during cardiopulmonary resuscitation: a systematic review and meta-analyses. BMC Pregnancy Childbirth. 2022 Feb 25;22(1):159.](https://www.zotero.org/google-docs/?ti1Mjy)

[51. Panchal AR, Bartos JA, Cabañas JG, Donnino MW, Drennan IR, Hirsch KG, et al. Part 3: Adult Basic and Advanced Life Support: 2020 American Heart Association Guidelines for Cardiopulmonary Resuscitation and Emergency Cardiovascular Care. Circulation. 2020 Oct 20;142(16_suppl_2):S366–468.](https://www.zotero.org/google-docs/?ti1Mjy)

[52. Jain V, Chari R, Maslovitz S, Farine D, Maternal Fetal Medicine Committee, Bujold E, et al. Guidelines for the Management of a Pregnant Trauma Patient. J Obstet Gynaecol Can JOGC J Obstet Gynecol Can JOGC. 2015 Jun;37(6):553–74.](https://www.zotero.org/google-docs/?ti1Mjy)

Abbreviations

ACLS Advanced Cardiovascular Life Support

ATLS Advanced Trauma Life Support

CL Cervical length

CPR Cardiopulmonary resuscitation

CS Cesarean section

ECV External cephalic version

ET Embryo transfer

IR Implantation rate

IUI Intrauterine insemination

IVC Inferior vena cava

MRI Magnetic resonance imaging

NPV Negative predictive value

PPROM Preterm prelabor rupture of membranes

PPV Positive predictive value

PR Pregnancy rate

PTB Preterm birth

RCT Randomized controlled trial

SFH Symphysis-fundal height

SHS Supine hypotensive syndrome
